# Systematics and phylogeny of the Zygodactylidae (Aves, Neognathae) with description of a new species from the early Eocene of Wyoming, USA

**DOI:** 10.7717/peerj.4950

**Published:** 2018-06-25

**Authors:** N. Adam Smith, Aj M. DeBee, Julia A. Clarke

**Affiliations:** 1 Campbell Geology Museum, Clemson University, Clemson, SC, USA; 2 Jackson School of Geosciences, University of Texas at Austin, Austin, TX, USA

**Keywords:** Avian evolution, Fossil birds, Stem passeriform, *Zygodactylus*, *Primozygodactylus*, *Eozygodactylus*, Green River Formation, *Zygodactylus grandei*, *Primoscens*

## Abstract

Zygodactylidae are an extinct lineage of perching birds characterized by distinct morphologies of the foot and wing elements. Although the clade has a complex taxonomic history, current hypotheses place Zygodactylidae as the sister taxon to Passeriformes (i.e., songbirds). Given the rather sparse fossil record of early passeriforms, the description of zygodactylid taxa is important for inferring potentially ancestral states in the largest radiation of living birds (i.e., the ∼6,000 species of extant passeriforms). Despite the exceptional preservation of many specimens and considerable species diversity in Zygodactylidae, the relationships among species have not been previously evaluated in a phylogenetic context. Herein, we review the fossil record of Zygodactylidae from North America and describe five new well-preserved fossils from the early Eocene Green River Formation of Wyoming. Two specimens are identified as representing a new species and the first records of the taxon *Zygodactylus* outside Europe. Anatomical comparisons with previously named taxa and the results of phylogenetic analysis including newly described specimens and previously named zygodactylid taxa provide the first hypothesis of the species-level relationships among zygodactylids. The monophyly of Zygodactylidae is supported in these new analyses. However, the monophyly of *Primozygodactylus* and the taxonomic distinction between *Zygodactylus* and *Eozygodactylus* remain unresolved and would likely benefit from the description of additional specimens.

## Introduction

Zygodactylidae Brodkorb, 1971 is an extinct, comparatively species-rich clade of enigmatic birds that possess derived morphological features associated with a perching habitus (Mayr, 2008, 2009, 2015). Zygodactylidae is primarily characterized by a zygodactyl conformation of the pedal phalanges—possessing a retroverted fourth toe and associated accessory trochlea on the distal end of the tarsometatarsus (Olson & Feduccia, 1979). Fossil zygodactylids are most abundant in Eocene deposits of North America and Europe, although proposed members of the clade are also known from the Oligocene and early Miocene of Europe (Mayr, 2008, 2009).

The systematic history of Zygodactylidae is complex (summarized in Table 1). Zygodactylidae have been allied with rollers (i.e., the clade refrred to as Coracii sensu Clarke et al., 2009, 2014; Olson & Feduccia, 1979), woodpeckers (Piciformes and allies; Brodkorb, 1971; Mayr, 1998), and most recently, perching birds (Passeriformes; Mayr, 2004, 2008, 2015). The results of a phylogenetic analysis of zygodactylid genera supported a monophyletic Zygodactylidae, comprising *Primozygodactylus*, *Primoscens*, and *Zygodactylus* (Mayr, 2008). However, the species-level interrelationships of described Zygodactylidae have not been previously evaluated in a phylogenetic analysis, and North American taxa have received relatively little attention in comparison with European taxa. Previously described taxa from North America comprise only a single species, *Eozygodactylus americanus* Weidig, 2010, known only from the Fossil Butte Member (FBM) of the Green River Formation (Weidig, 2010). In contrast, eight zygodactylid species placed in three genera (*Zygodactylus*, *Primozygodactylus*, *Primoscens*) are known from Europe (Mayr, 2009, 2015).

**Table 1:** Summary of previous taxonomic assessments regarding the affinities of Zygodactylidae and zygodactylid taxa.

Taxon	Previous referral	Reference
*Zygodactylus ignotus*	Indeterminate	Ballmann (1969a)
*Zygodactylus grivensis*	cf. Passeriformes	Ballmann (1969b)
*Zygodactylus*	Piciformes	Harrison & Walker (1977)
*Zygodactylus*	Piciformes	Simpson & Cracraft (1981)
“Primoscenidae”	Passeriformes	Harrison & Walker (1977)
“Primoscenidae”	Piciformes	Mayr (1998)
“Primoscenidae”	Passeriformes	Harrison & Walker (1977)
Zygodactylidae	Sister to Passeriformes	Mayr (2008, 2015)
Zygodactylidae + “Primoscenidae”	Sister to Passeriformes	Mayr (2004)

The systematic relationships of five new zygodactylid specimens from the Eocene of North America are evaluated with other specimens referred to previously recognized zygodactylid taxa to investigate the monophyly of zygodactylid subclades and the relationships of named zygodactylid species. By assessing species-level diversity and relationships in this clade, we are able to generate new data regarding paleobiogeographical patterns of Zygodactylidae.

### Taxonomic history of Zygodactylidae

The clade name Zygodactylidae was originally proposed by Brodkorb (1971) for fragmentary material from the lower Miocene of Germany and the middle Miocene of France. Zygodactylidae, sensu Brodkorb (1971), included only *Zygodactylus ignotus* and *Z. grivensis* (Ballmann, 1969a, 1969b). Despite the morphological similarities between Zygodactylidae and new species described in the following years, the contents of Zygodactylidae remained stable until 2008, when the taxon Primoscenidae Harrison & Walker, 1977, was deemed a junior synonym of Zygodactylidae by Mayr (2008). As a result, *Primoscens minutus* Harrison & Walker, 1977, *Primozygodactylus major* Mayr, 1998, *Primozygodactylus danielsi* Mayr, 1998, and *Primozygodactylus ballmanni* Mayr, 1998 are now considered part of Zygodactylidae. This taxonomic revision was based on the shared the presence of three characters: a pronounced trochlea accessoria; elongate tarsometatarsus; and pronounced unfused intermetacarpal process (Mayr, 1998).

The name “Primoscenidae” was originally coined in reference to a species identified from an isolated carpometacarpus (*Primoscens minutus* Harrison & Walker, 1977) from the early Eocene London Clay Formation. This specimen was commented on by Harrison & Walker (1977), who noted its prominent intermetacarpal process and general similarities with Passeriformes. *Primoscens minutus* was the only species assigned to Primoscenidae when Mayr (1998) referred 18 additional specimens to “Primoscenidae,” although isolated elements in a private collection from the London Clay had been previously proposed to belong to Primoscenidae (pers. comm. from Daniels cited by Feduccia, 1999). The specimens referred by Mayr (1998) are from the middle Eocene deposits of Messel, Germany, as well as the early Eocene Green River Formation in North America and the late Paleocene/early Eocene Fur Formation of Denmark. Three new “Primoscenidae” species, *Primozygodactylus danielsi*, *Primozygodactylus major*, and *Primozygodactylus ballmanni*, were identified by Mayr (1998) and only later referred to Zygodactylidae (Mayr, 2008).

More recently, *Z. luberonensis* Mayr, 2008, a new species of Zygodactylidae, was described based on a nearly complete specimen found in the early Oligocene lacustrine deposits of the Luberon area in Southern France (Mayr, 2008). This taxon was found to possess not only the zygodactyl foot typical of “primoscenids” and zygodactylids, but shared with “Primoscenidae” (i.e., *Primoscens + Primozygodactylus*), amongst other characters, a ventrally-displaced insertion of the *m. brachialis* on the humerus, a well-developed dorsal supracondylar process, and a well-developed intermetacarpal process that was not fused to metacarpal III. These similarities were determined by Mayr (2008) to warrant synonymization of “Primoscenidae” with Zygodactylidae, rendering “Primoscenidae” a junior synonym of Zygodactylidae. However, the accessory trochlea on the tarsometatarsus was proposed to be more bulbous and distally extended in *Zygodactylus* (now with an Eocene–Miocene distribution) than in *Primoscens* and *Primozygodactylus*, suggestive of distinct subclades within Zygodactylidae.

Zygodactylidae have been previously allied with several extant avian taxa. Despite the apparent zygodactyl condition of *Z. grivensis* and *Z. ignotus* (both known only from distal tarsometatarsal and tibiotarsal fragments), Ballmann (1969a, 1969b) was unsure of the appropriate taxonomic assignment for these taxa and doubted they were most closely related to the extant clade Piciformes, which also have a zygodactyl foot condition. Instead, Ballmann (1969a, 1969b) suggested possible passeriform affinities for *Z. ignotus*. Subsequently, the assignment of Zygodactylidae to Piciformes was proposed by Harrison & Walker (1977), but these authors also considered the “primoscenid” *P. minutus* to possibly represent a basal passeriform. *Z. grivensis* was tentatively suggested to be a part of Piciformes by Simpson & Cracraft (1981) based on the presence and structure of the accessory trochlea. These authors argued that the size and position of the accessory trochlea was more similar to the piciform subclade Pici than to Galbulidae or to Bucconidae, and suggested *Zygodactylus* might be a part of Pici. They also noted “[t]he morphology of *Z. grivensis* is unique, however, and an assignment of this form to a particular suborder [within Piciformes] is difficult” (Simpson & Cracraft, 1981: 492). In 1998, Mayr specifically considered *Zygodactylus* a sister taxon to Pici, and “Primoscenidae” as the sister taxon to that clade within crown-group Piciformes. It should be noted, that Mlíkovský’s (1996) referral of extinct taxa *Procolius* and *Quercypsitta* to Zygodactylidae has not been supported by subsequent researchers and these taxa appear unrelated to each other or to other proposed zygodactylid species (Mayr, 2009).

The phylogenetic analysis of Mayr (2004) focused on the relationships of “Primoscenidae” and Zygodactylidae. That analysis largely examined the systematic position of Zygodactylidae within Aves and used morphological characters scored for 17 supraspecific taxa representing major avian subclades (Mayr, 2004). Resultant tree topologies from that analysis indicated a sister-taxon relationship between Passeriformes and a clade composed of Zygodactylidae and “Primoscenidae” (prior to the recognition of “Primoscenidae” as a junior synonym of Zygodactylidae by Mayr, 2008). The cladistic matrix of Mayr (2004) was reanalyzed (Mayr, 2008) with additional morphological data from *Z. luberonensis*, and included terminals for both *Zygodactylus* and *Primozygodactylus*. The results of that analysis provided further justification for a sister-taxon relationship between Passeriformes and Zygodactylidae. Subsequently, a phylogenetic analysis with relatively dense outgroup sampling across Aves also placed Zygodactylidae (*Zygodactylus* + *Primozygodactylus*) as the sister taxon to Passeriformes (Mayr, 2015). Although Passeriformes represent the most speciose clade of living birds (∼6,000 species), the early fossil record of passeriforms is limited to a handful of proposed Paleogene exemplars (Boles, 1997a; Mayr, 2009; Bocheński et al., 2011, 2013). Thus, additions to our knowledge of Zygodactylidae, the proposed sister taxon to Passeriformes, have the potential to provide insights regarding the diversity patterns and inference of ancestral morphological conditions in stem Passeriformes.

### Geologic setting

**Figure 1:**
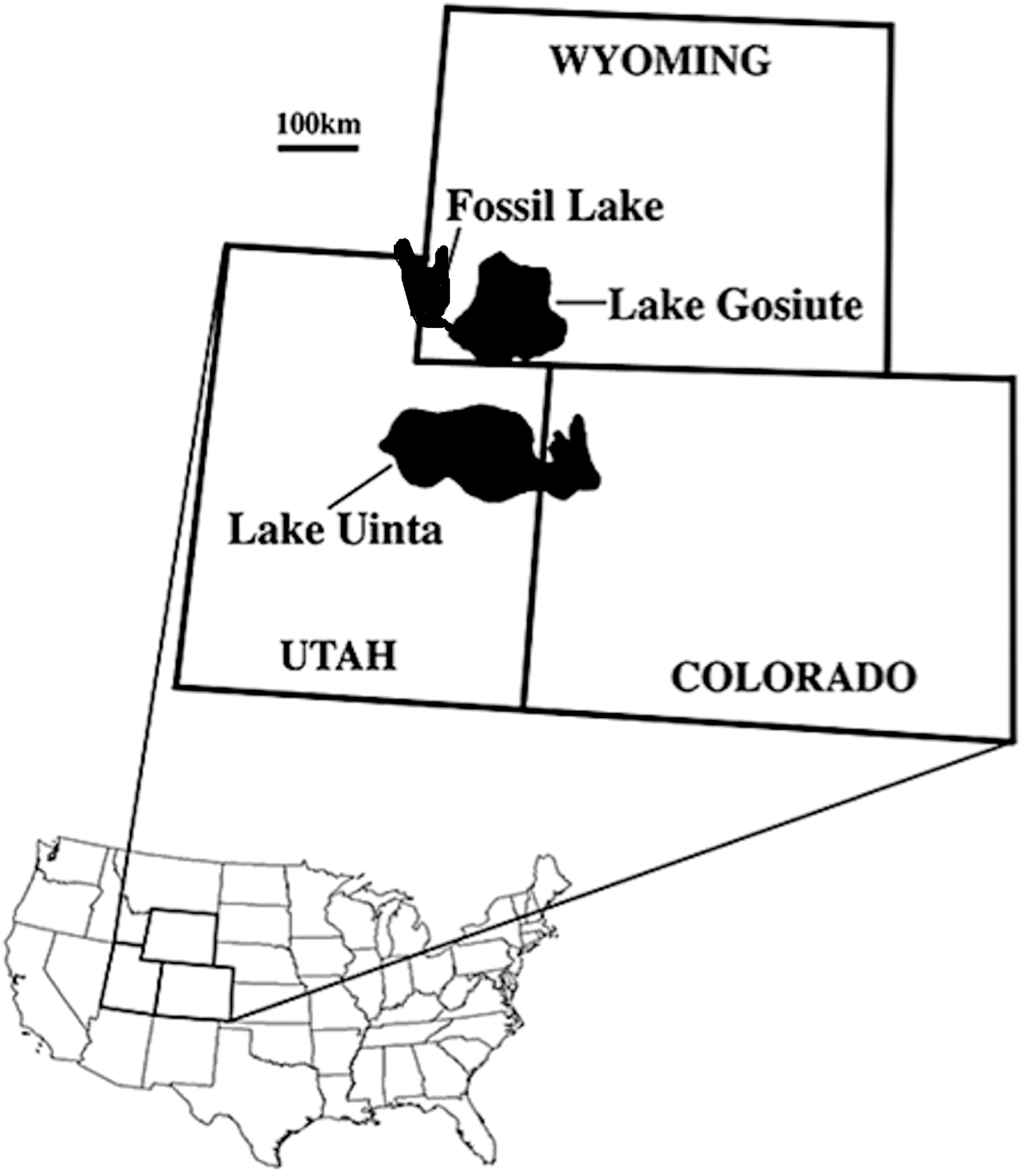
Map of the USA showing the location of the Green River lake system during the late early Eocene (∼52 Ma; from Ksepka & Clarke, 2010b; reprinted with permission of the authors).

We describe five zygodactylid fossils from the FBM of the early Eocene (∼52 Ma, Ypresian) Green River Formation of Wyoming (Fig. 1). The lacustrine deposits of the Green River Formation are from three ancient lakes that spanned portions of present-day Wyoming, Colorado, Idaho, and Utah (Fig. 1; Grande, 2013). The age of the Green River Formation spans the late Paleocene to the middle to late Eocene, and the FBM possesses the most species-rich paleontological record known from North American Tertiary aquatic communities (Grande, 1984, 1994, 2013). The FBM includes deposits of the smallest and most short-lived of the Green River lakes, Fossil Lake, which is located in at the junction of Wyoming, Idaho, and Utah (Grande, 2013). The FBM is bounded above by a potassium feldspar rich tuff, which was dated to 51.66 ± 0.20 Ma with 40Ar/39Ar spectrometry (Smith, Carroll & Singer, 2008; Smith et al., 2010), providing constraint on the minimum age for the unit. Estimates of the rate of deposition suggest a geologically short timeframe of several thousand years for the FBM, whereas the timeframe for deposition of the entirety of the Green River Formation spans from 90,000 to 180,000 years (Grande & Buchheim, 1994; Grande, 2013).

The Green River Formation has yielded voluminous collections of trace fossils, non-vertebrates and some spectacular articulated specimens of vertebrates including multiple species of fish, amphibians, crocodiles, turtles, squamates, mammals, and birds (Grande, 1994, 2013; Grande & Buchheim, 1994). Most of the known avian diversity in the Green River Formation is from the FBM (Grande, 1994, 2013) and includes the most well-preserved and taxonomically diverse avifauna from the Eocene of North America (Grande, 2013). Three-dimensionally preserved skeletons, flattened or crushed body fossils, as well as feathers and feather impressions have been recovered from the FBM. Fossil birds from the FBM include stem representatives of mousebirds (Coliiformes; Ksepka & Clarke, 2010a), rollers (Coracii; Clarke et al., 2009, 2014; Ksepka & Clarke, 2010b), parrots (Pan-Psittaciformes; Ksepka, Clarke & Grande, 2011), frogmouths (Podargiformes; Nesbitt, Ksepka & Clarke, 2011), galliforms (Galliformes; Mayr & Weidig, 2004; Weidig, 2010), crane-like birds (Gruiformes; Weidig, 2010); paleognaths (Lithornithiformes; Grande, 2013), duck-like birds (Anseriformes; Clarke pers. comm. in Grande, 2013), frigatebirds (Pelecaniformes; Olson, 1977; Olson & Matsuoka, 2005), ibis-like birds (Ciconiiformes; Smith, Grande & Clarke, 2013) and an array of tentatively referred taxa and as-yet-undescribed forms (Grande, 2013; Ksepka & Clarke, 2010b; N. Adam Smith, 2015, personal observation).

The ever-growing diversity of birds documented from the Green River Formation provides the earliest window into the Cenozoic avifauna of North America (Grande, 2013). The only comparable Eocene avifauna is that of Messel, Germany (see Mayr, 2009 and references therein). Ongoing research focused on these relatively contemporaneous avifaunas continues to provide evolutionary insights into the early stages of the radiation of the avian crown clade and has resulted in the recognition of some surprising similarities between these geographically disjunct communities. Herein, we add an additional species of Zygodactylidae to the North American fossil record, document the first occurrence of the taxon *Zygodactylus* from outside of Europe, and discuss the biogeographical implications of relatively coeval populations of zygodactylids from the early Eocene of North America and Europe.

## Materials and Methods

### Anatomical, geological, and taxonomic conventions

Osteological terms used are English equivalents of the Latin nomenclature summarized in Baumel & Witmer (1993). Unless otherwise stated, measurements represent the maximum linear length of skeletal elements along the longitudinal axis in millimeters. Measurements were taken with digital calipers to the nearest 1/10th of a millimeter. Measurements of three specimens (USNM 299821, WDC-CGR-014, NAMAL 2000-0217-004) are those reported by Weidig (2010). Measurements of *Nestor notabilis* are those of Livezey (1992). Measurements of all other sampled specimens were taken directly from holotype and referred specimens. Ages of geologic time intervals are based on the International Geologic Timescale (International Commission on Stratigraphy, 2017). With the exception of species binomials, all taxonomic designations (e.g., Zygodactylidae) are clade names as defined by the PhyloCode (Dayrat et al., 2008, Cantino & de Queiroz, 2010) and are not intended to convey rank under the Linnaean system of nomenclature.

The electronic version of this article in portable document format will represent a published work according to the International Commission on Zoological Nomenclature (ICZN), and hence the new names contained in the electronic version are effectively published under that Code from the electronic edition alone. This published work and the nomenclatural acts it contains have been registered in ZooBank, the online registration system for the ICZN. The ZooBank LSIDs (Life Science Identifiers) can be resolved and the associated information viewed through any standard web browser by appending the LSID to the prefix http://zoobank.org/. The LSID for this publication is: 8F4D9D54-AFE5-4A79-8A49-FC804996DB5E. The online version of this work is archived and available from the following digital repositories: PeerJ, PubMed Central, and CLOCKSS.

### Taxon and character sampling

A total of five specimens from the FBM of the Green River Formation are described herein. These specimens are: FMNH PA 726; FMNH PA 757; FMNH PA 770; UWGM 40705; and UWGM 40363. Anatomical comparisons were made with the following specimens of Zygodactylidae that represent all previously described species in the clade: *Primozygodactylus eunjooae* (SMF-ME 1074, holotype; SMF-ME 11537a); *Primozygodactylus major* (SMF-ME 799a+b, SMF-1758a+b (holotype)); *Primozygodactylus danielsi* (SMF-ME 2522a+b (holotype), SMF-ME 1817, HLMD-Me 15550a+b, HLMD-Me 10206a+b; SMF-ME 10835a); *Primozygodactylus ballmanni* (SMF-ME 2108 (holotype), HLMD-Me 15396); *Primozygodactylus quintus* (SMF-ME 11091a+b, SMF-ME 10794a+b); *Primozygodactylus longibrachium* (SMF-ME 11171a+b); *Primoscens minutus* (BMNH A 4681, holotype); *Z. luberonensis* (SMF-Av 519, holotype); *Z. ignotus* (BSP 18164, holotype); *Z. grivensis* (FSL 151, holotype); and *E. americanus* (USNM 299821 (holotype) and WDC-CGR-014 (paratype)). Morphological characters of *Primoscens minutus*, *Primozygodactylus quintus*, *Primozygodactylus longibrachium*, *Z. ignotus*, *Z. grivensis*, *Jamna szybiaki*, and *Cyrilavis colburnorum* were evaluated from published sources (Mayr, 2004, 2008, 2017; Bocheński et al., 2011; Ksepka, Clarke & Grande, 2011). Morphological characters of *E. americanus* were evaluated from photos of the holotype specimen provided by a colleague and from the original description of that taxon (Weidig, 2010). All other sampled taxa were evaluated directly from holotype and referred specimens.

### Phylogeny estimation

A total of 20 specimens of Zygodactylidae and six outgroup taxa were evaluated (see Appendix I for morphological character descriptions and scorings). Morphological scorings for both specimens of *Z. grandei* (holotype, FMNH PA 726; referred, UWGM 40705) were combined to form a single terminal. Likewise, all three specimens of *E. americanus* (holotype, USNM 299821; paratype, WDC-CGR-014; referred, FMNH PA 770) were combined into a single terminal for the purposes of phylogenetic analysis. Outgroup taxa were chosen to include clades previously hypothesized to be closely related to Zygodactylidae. Dense sampling of the comparatively species rich crown-clades of Passeriformes (perching birds), Psittaciformes (parrots) and Piciformes (woodpeckers and allies) was outside the scope of this analysis, and supraspecific terminals were employed for these groups. Passeriformes scorings were obtained from two suboscines (*Tyrannus tyrannus*, *Thamnophilus caerulescens*), and two oscines (*Corvus bracnyrunchus*, *Menura novahollandiae*). The Rifleman, *Acanthisitta chloris,* was scored as a separate terminal, due to its sister-group relationship to the clade containing oscines and suboscines (Barker, Barrowclough & Groth, 2002; Ericson et al., 2006; Suh et al., 2011; Hackett et al., 2008; Jarvis et al., 2014). The Oligocene taxon *J. szybiaki* (Bocheński et al., 2011) was included to represent the putative stem lineage of Passeriformes. Piciformes is represented by a supraspecific terminal based on evaluation of the following taxa: *Dryocopus pileatus*, *Colaptes auratus*, *Galbula ruficada* and *Chelidoptera tenebrosa*. A supraspecific terminal for Psittaciformes was based on scorings representing *N. meridionalis* and *N. notabilis*, given their basal placement within that clade (Miyaki et al., 1998; Wright et al., 2008; Ksepka, Clarke & Grande, 2011). The Eocene taxon *C. colburnorum* (Ksepka, Clarke & Grande, 2011) was included to represent the stem lineage of Psittaciformes.

A total of 50 discrete morphological characters were coded for phylogenetic analysis in Mesquite v3.4 (Maddison & Maddison, 2018). As noted in Appendix I, characters were drawn in part from observations by Ashley (1941), Zelenkov (2007), Manegold (2008), Mayr (2004, 2008, 2015), Weidig (2010), and Bocheński et al. (2011). A total of six multistate characters appear to form natural morphoclines and were ordered (Slowinski, 1993; Appendix I, characters 13, 18, 20, 21, 27, 42). Multistate scorings resulting from variation among exemplar taxa evaluated for the supraspecific terminals were treated as polymorphic. Phylogenetic analyses used a maximum parsimony estimator in PAUP*4.0a159 (Swofford, 2002). Tree searches employed the following parameters: branch-and-bound search strategy; tree bisection-reconnection branch swapping; random starting trees; all characters equally weighted; minimum length branches = 0 collapsed. Descriptive tree statistics including consistency index, retention index, and bootstrap support values from 1,000 replicates (100 random sequence additions per replicate) were computed using PAUP*4.0a159 (Swofford, 2002). Resultant trees were rooted with the supraspecific terminal representing Piciformes based on the congruent, molecular-based hypotheses of avian relationships of Hackett et al. (2008) and Jarvis et al. (2014).

## Systematic Paleontology

**Aves** Linnaeus C. von, 1758
**Neognathae** Pycraft, 1900
**Zygodactylidae** Brodkorb, 1971

### Emended diagnosis

The following suite of four previously identified characters from Mayr (2008) and one newly identified character are diagnostic for Zygodactylidae. The wordings of the following four character descriptions were modified slightly for clarity. The carpometacarpus has a distinct intermetacarpal process that is unfused to metacarpal III (Appendix I, characters 29, 30) and a distinct protuberance on the anterior margin of metacarpal II close to its midpoint (Appendix I, character 28; i.e., “dentiform process” of Mayr, 2004). *Eozygodactylus,* however, does not possess a dentiform process, representing a reversal in that taxon. Parrots, falcons and *Seriama* (i.e., putative near outgroups to Pan–Passeriformes) lack an intermetacarpal process. Thus, the possibility that the primitive state (i.e., possession of and unfused process) is retained in Zygodactylidae should be considered. The tarsometatarsus distinctly exceeds the humerus in length (Appendix I, character 42). However, it should be noted that this character is variably present in crown passerines, and the stem passerine *Wieslochia*, but is not preserved in *Jamna*. The wide distribution of this character (present in wading birds, hummingbirds, and hawks for example) suggests its potential homoplastic nature. Thus, utility of this character may be diminished as additional stem passeriforms are identified and its status as a synapomorphy should be reevaluated based upon recovery of additional fossils. The furcula possesses a broad, subtriangular omal end (Appendix I, character 9). A ventrally bowed jugal bar (Appendix I, character 3) was determined from the present study to be a local synapomorphy of the clade. Mayr (2008) also considered the presence of a hypotarsus with only two bony canals, well-developed cnemial crests on the tibiotarsus, and the presence of a lateral plantar crest on the tarsometatarsus to be diagnostic of Zygodactylidae, but those features are highly homoplastic within Aves (Livezey & Zusi, 2006, 2007) and are of more limited utility.

### Taxonomic remarks

As proposed by Mayr (2008), “Primoscenidae” is considered a junior synonym of Zygodactylidae herein.

### Taxa included

Genera: *Zygodactylus* Ballmann, 1969; *Primoscens* Harrison & Walker, 1977; *Primozygodactylus* Mayr, 1998; *Eozygodactylus* Weidig, 2010.

Species: *Z. ignotus* Ballmann, 1969; *Z. grivensis* Ballmann, 1969; *Z. luberonensis* Mayr, 2008; *Primoscens minutus* Harrison & Walker, 1977; *Primozygodactylus danielsi* Mayr, 1998; *Primozygodactylus ballmanni* Mayr, 1998; *Primozygodactylus major* Mayr, 1998; *Primozygodactylus eunjooae* Mayr & Zelenkov, 2009; *Primozygodactylus quintus* Mayr, 2017; *Primozygodactylus longibrachium* Mayr, 2017; *E. americanus* Weidig, 2010; *Z. grandei* sp. nov.

***Zygodactylus*** Ballmann, 1969

**Type Species:** *Zygodactylus ignotus* Ballmann, 1969.

**Included Taxa:** *Zygodactylus grivensis* Ballmann, 1969; *Z. luberonensis* Mayr, 2008; *Z. grandei* sp. nov.

**Emended Diagnosis:** *Zygodactylus* is characterized by a unique combination of characters, including proposed autapomorphies (Mayr, 2008), which are designated by an “*” below. There is a distinct convexity on the lateral margin of the tarsometatarsus just proximal to the trochlea of metatarsal IV (Appendix I, character 39*), and a bulbous and distally elongate accessory trochlea on metatarsal IV (Appendix I character 45*). The coracoid is narrow and elongate, and the procoracoid process is reduced (Appendix I, character 14). The humerus possesses a tuberculate dorsal supracondylar process separated from the humeral shaft by a small notch (Appendix I, characters 19, 20). Metacarpal III extends distally, to a point well beyond metacarpal II (Appendix I, character 33). Character 39 was an autapomorphy of *Zygodactylus* proposed by Mayr (2008). It is absent in all taxa examined for this study save *Z. grivensis* and FMNH PA 726, supporting this assessment and placement the new specimen in *Zygodactylus*. This feature is absent in all taxa assigned to *Primozygodactylus* and *Primoscens*, however, the relevant region is not preserved in *Z. ignotus* or *E. americanus* and thus, cannot be assessed for those taxa. Character 45 was previously proposed to be an autapomorphy of *Zygodactylus* by both Ballmann (1969a, 1969b) and Mayr (2004).

***Zygodactylus grandei*** new species
(Figs. 2, 3C, 4–7, 8C, 9A; Tables 2–5)

**Figure 2:**
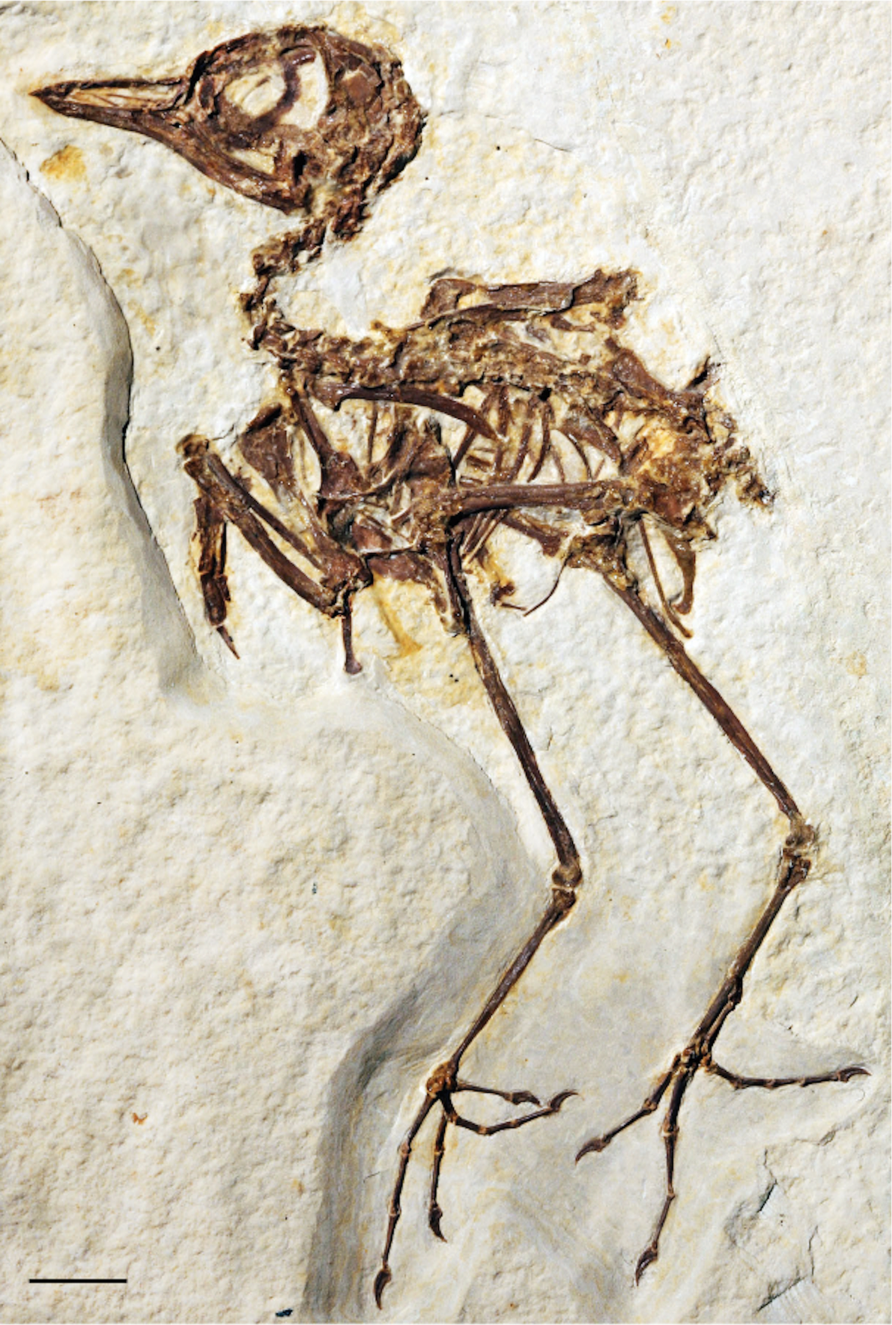
The holotype specimen of *Zygodactylus grandei* sp. nov. (FMNH PA 726) from the Green River Formation of Wyoming. Shown in left lateral view. Scale bar equals 1 cm.

**Figure 3:**
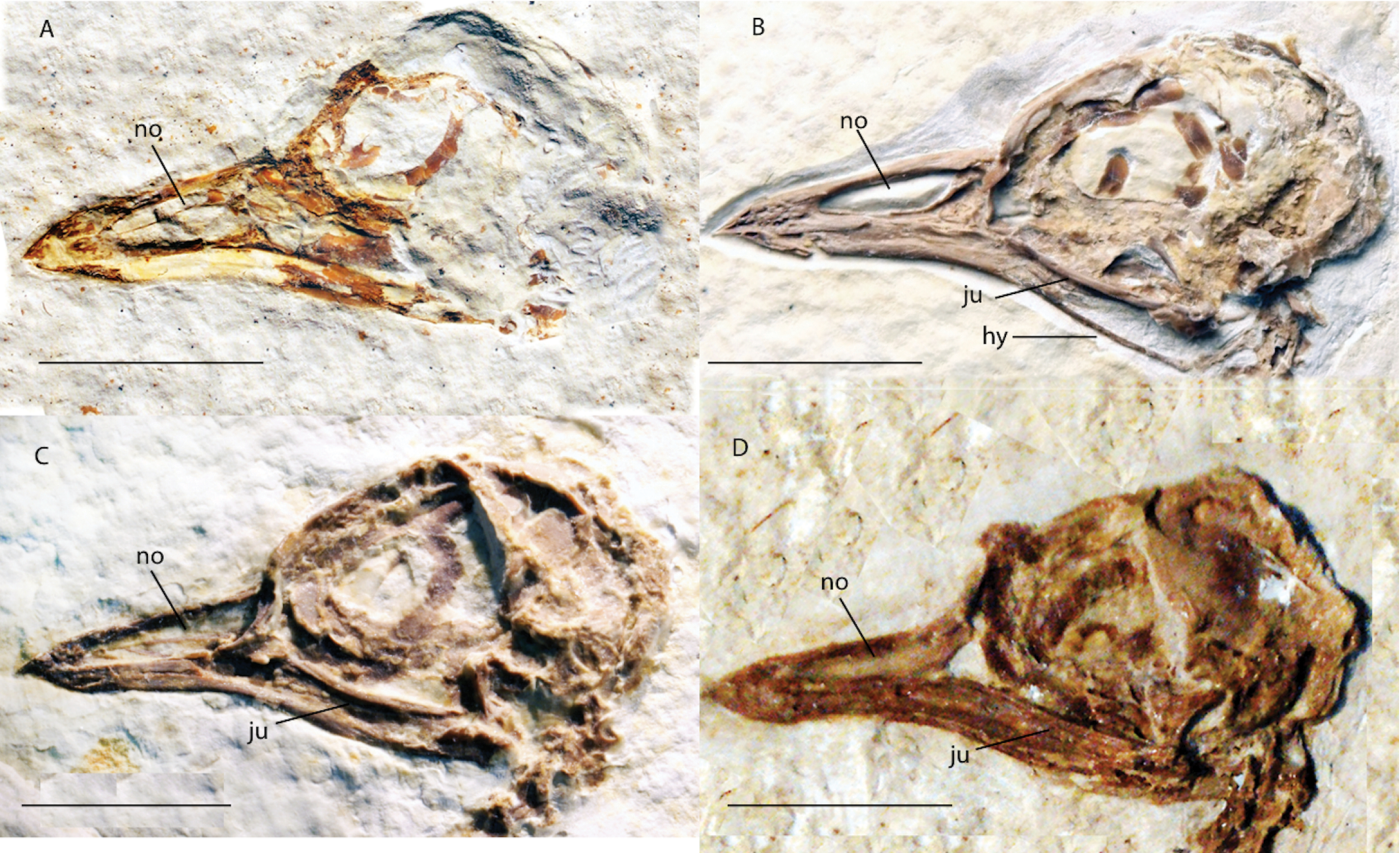
Crania of Green River Formation specimens referred to Zygodactylidae shown in left lateral view. (A) UWGM 40363, referred to Zygodactylidae gen. et sp. indet.; (B) FMNH PA 757, referred to Zygodactylidae gen. et sp. indet.; (C) FMNH PA 726, *Zygodactylus grandei*; (D) USNM 299821, *Eozygodactylus americanus*. Anatomical Abbreviations: hy, hyoid; ju, jugal; no, narial opening. Scale bars equal 1 cm.

**Figure 4:**
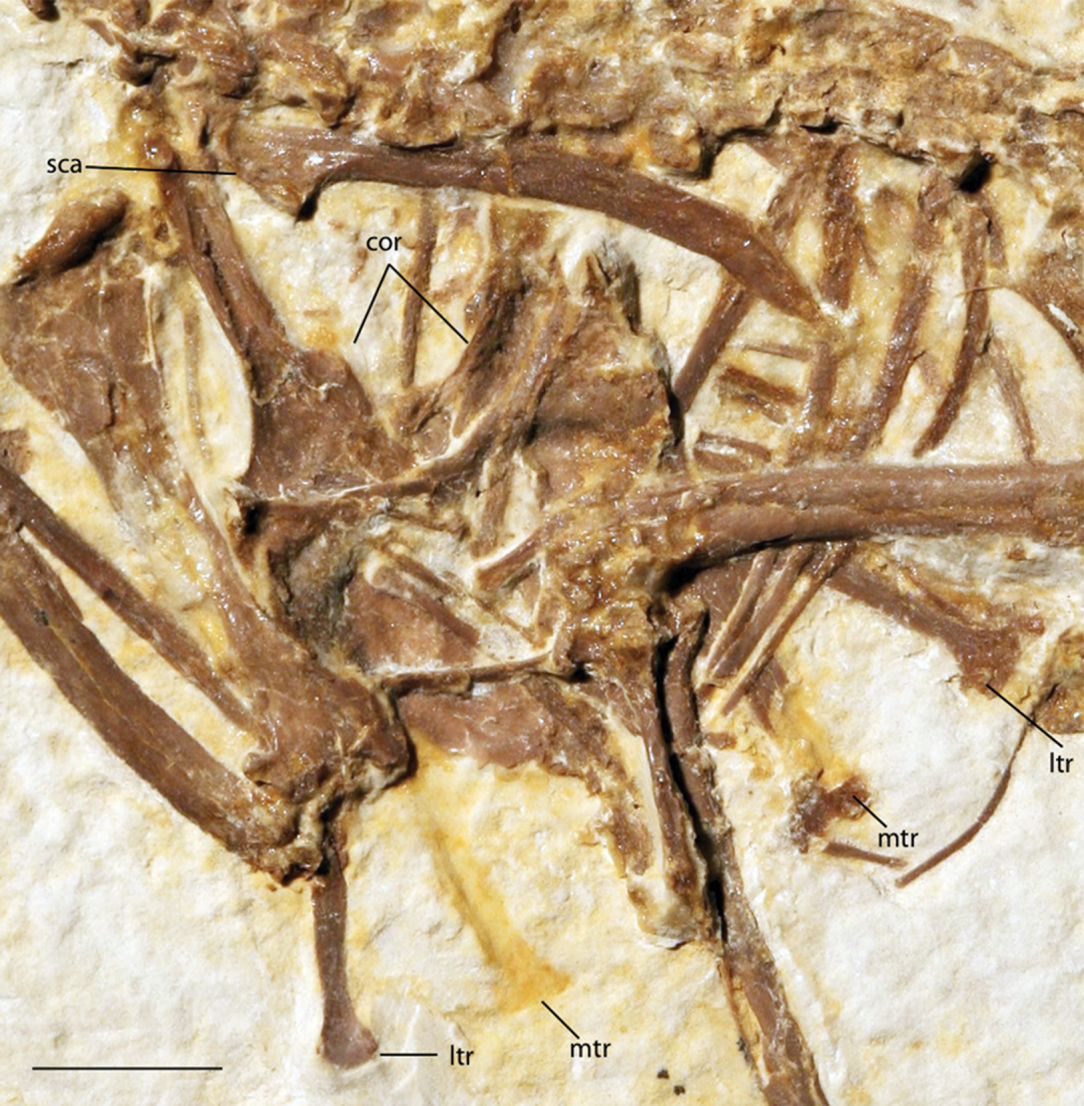
Pectoral girdle of FMNH PA 726, *Zygodactylus grandei*. Anatomical Abbreviations: cor, coracoid; ltr, lateral trabecula; mtr, medial trabecula; sca, scapula. Scale bar equals 1 cm.

**Figure 5:**
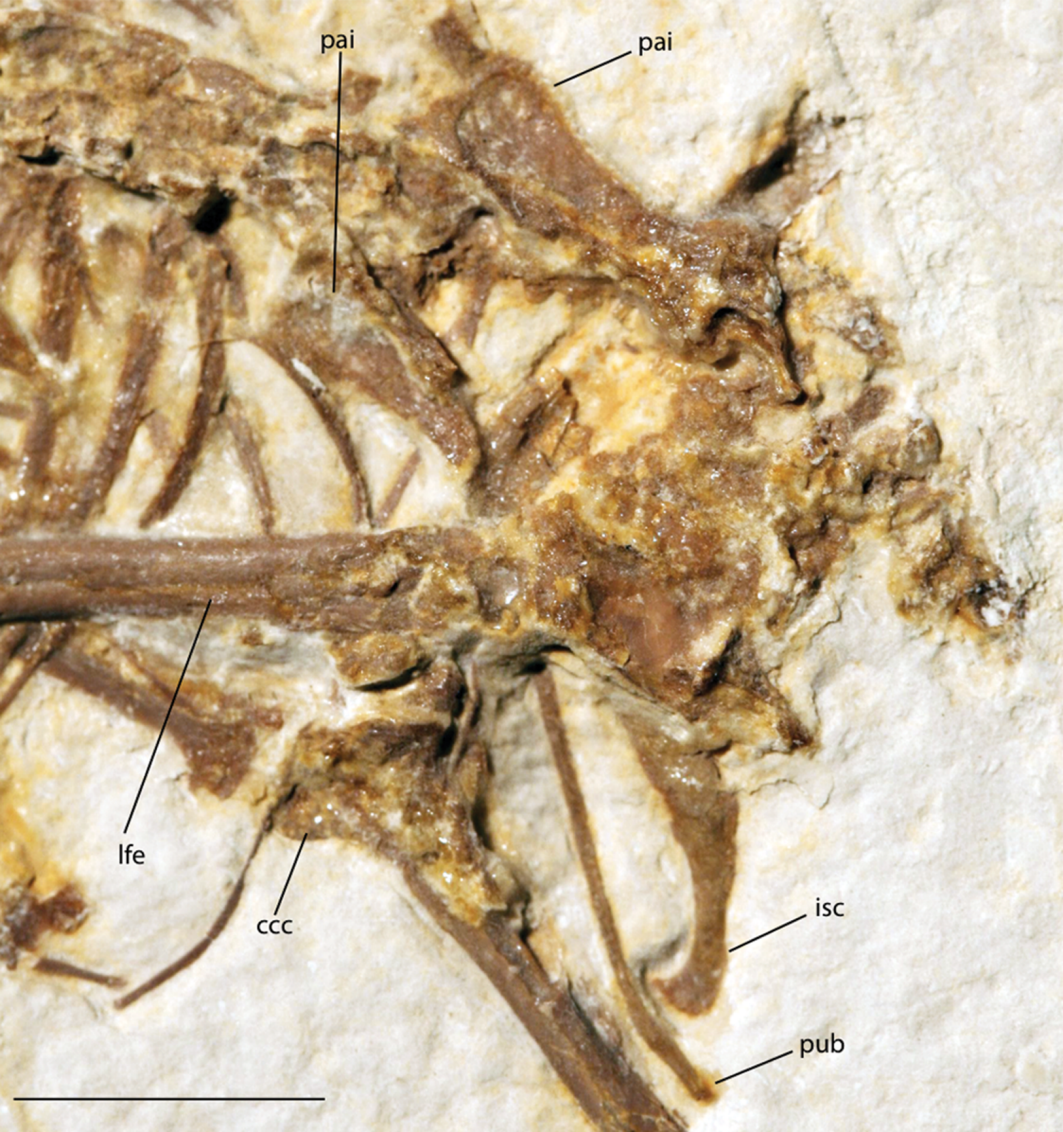
Pelvic girdle of FMNH PA 726, *Zygodactylus grandei*. Anatomical Abbreviations: ccc, cranial cnemial crest; isc, ischium; lfe, left femur; pai, preacetabular ilium; pub, pubis. Scale bar equals 1 cm.

**Figure 6:**
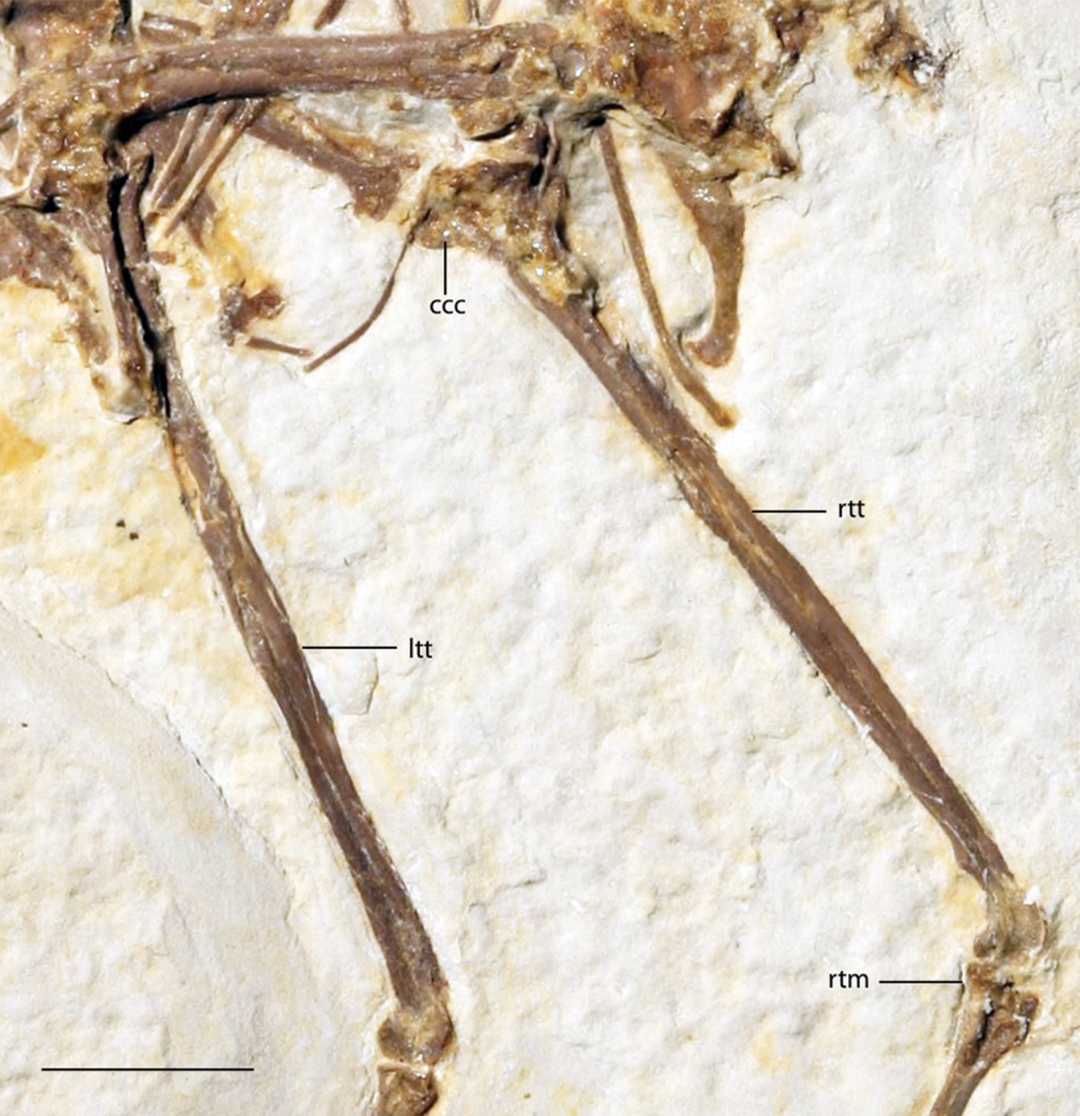
Tibiotarsi of FMNH PA 726, *Zygodactylus grandei*. Anatomical Abbreviations: ccc, cranial cnemial crest; itt, left tibiotarsus; rtm, right tarsometatarsus; rtt, right tibiotarsus. Scale bar equals 1 cm.

**Figure 7:**
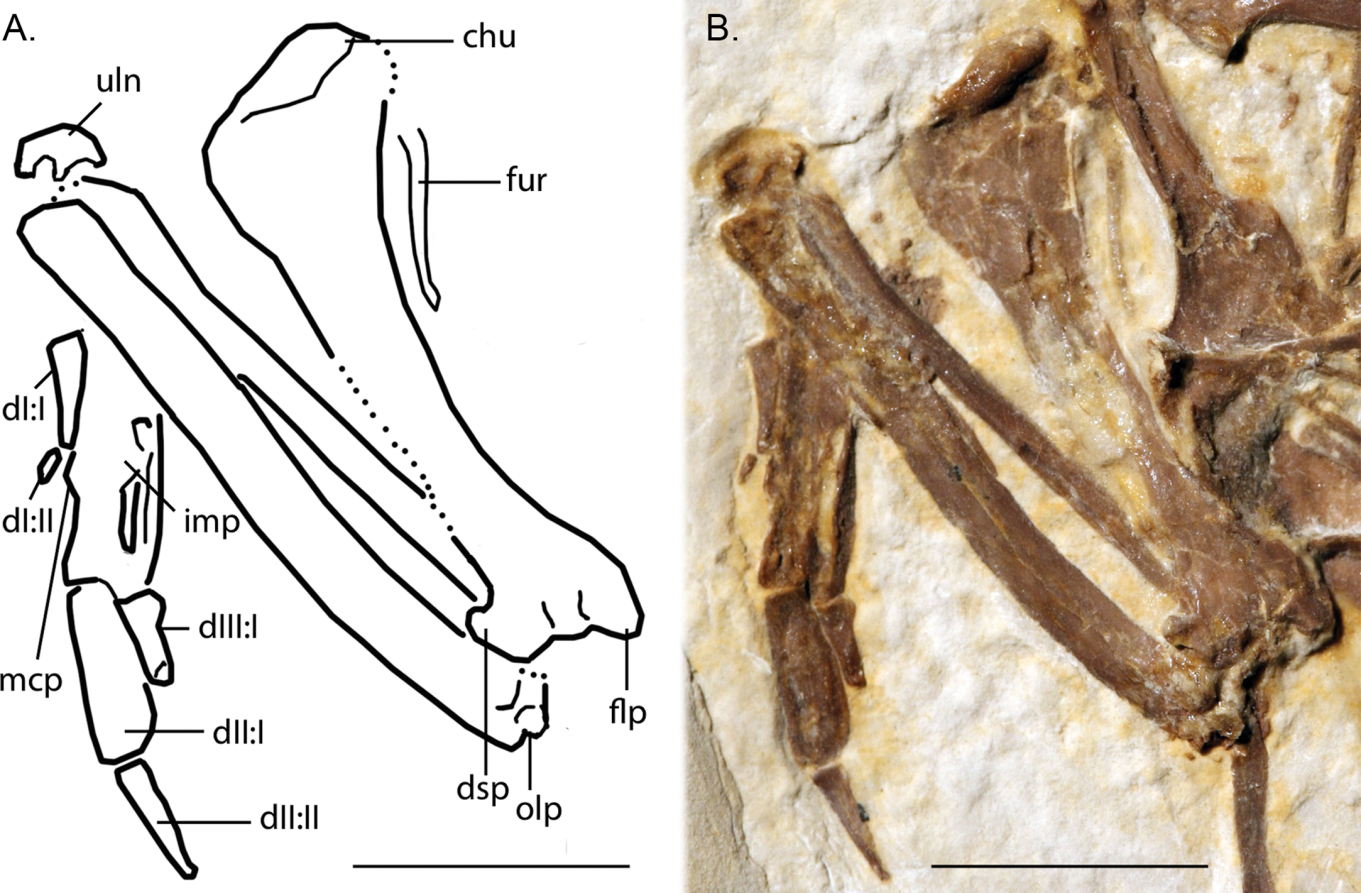
Line-drawing and photo of the left forelimb of FMNH PA 726, *Zygodactylus grandei*. Note the distinct dorsal supracondylar process and large intermetacarpal process. Anatomical Abbreviations: chu, caput humerus; dI:I, manual digit one, phalanx one; dI:II, manual digit one, phalanx two; dIII:I, manual digit three, phalanx one; dII:I, manual digit two, phalanx one; dII:II, manual digit two, phalanx two; dsp, dorsal supracondylar process; flp, flexor process; mpr, metacarpal protuberance (“dentiform process” of Mayr, 1998); olp, olecranon process; uln, ulnare. Scale bar equals 1 cm. Line drawing by A. Debee.

**Figure 8:**
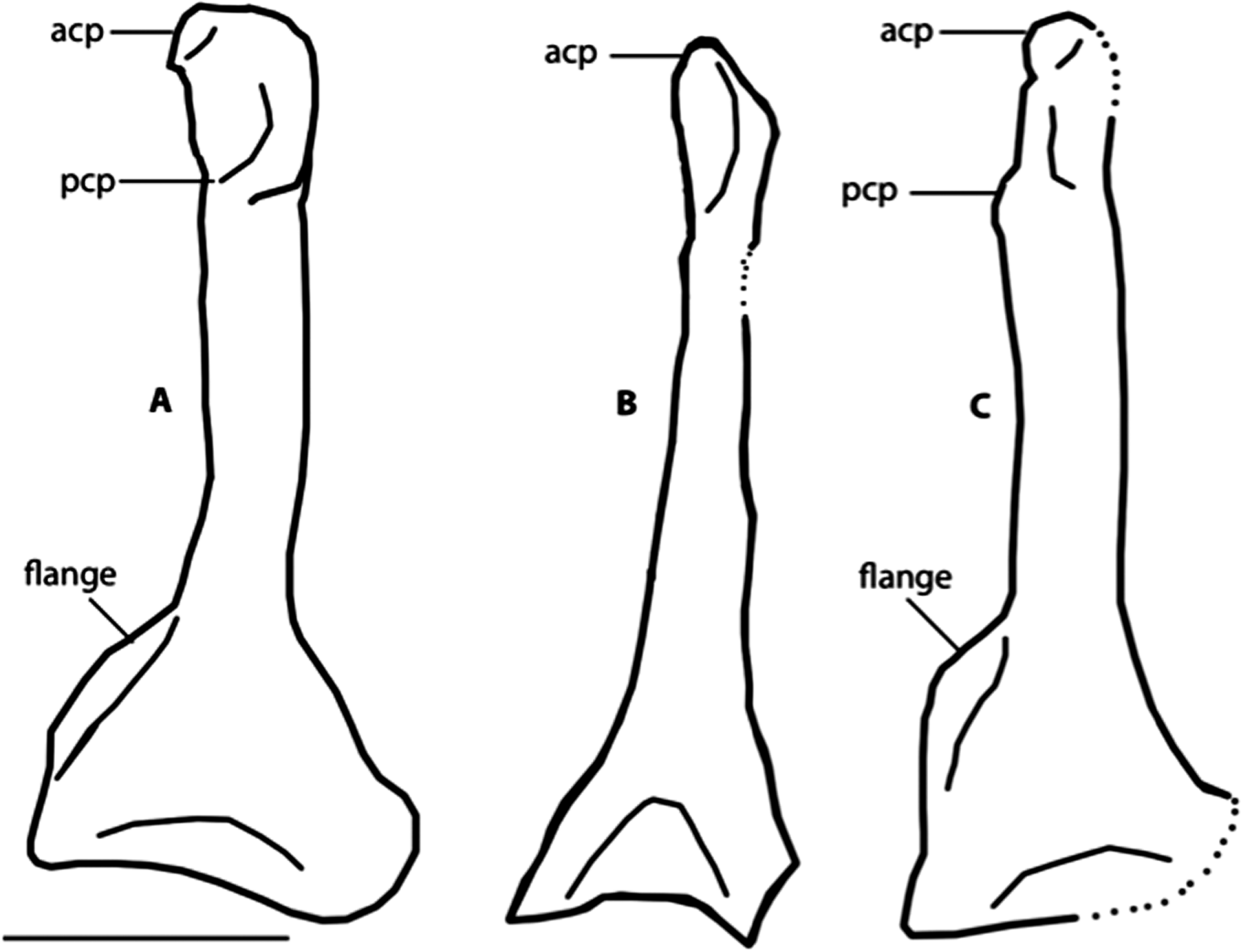
Dorsal view of right coracoids from: (A), *Primozygodactylus danielsi*, SMF 2552; (B) *Zygodactylus luberonensis*, SMF Av 519; and C, FMNH PA 726, *Zygodactylus* grandei. Note flange on medial side of coracoid. Anatomical Abbreviations: acp, acrocoracoid process; prp, procoracoid process. Scale bar equals 5 mm. Line drawing by A. Debee.

**Figure 9:**
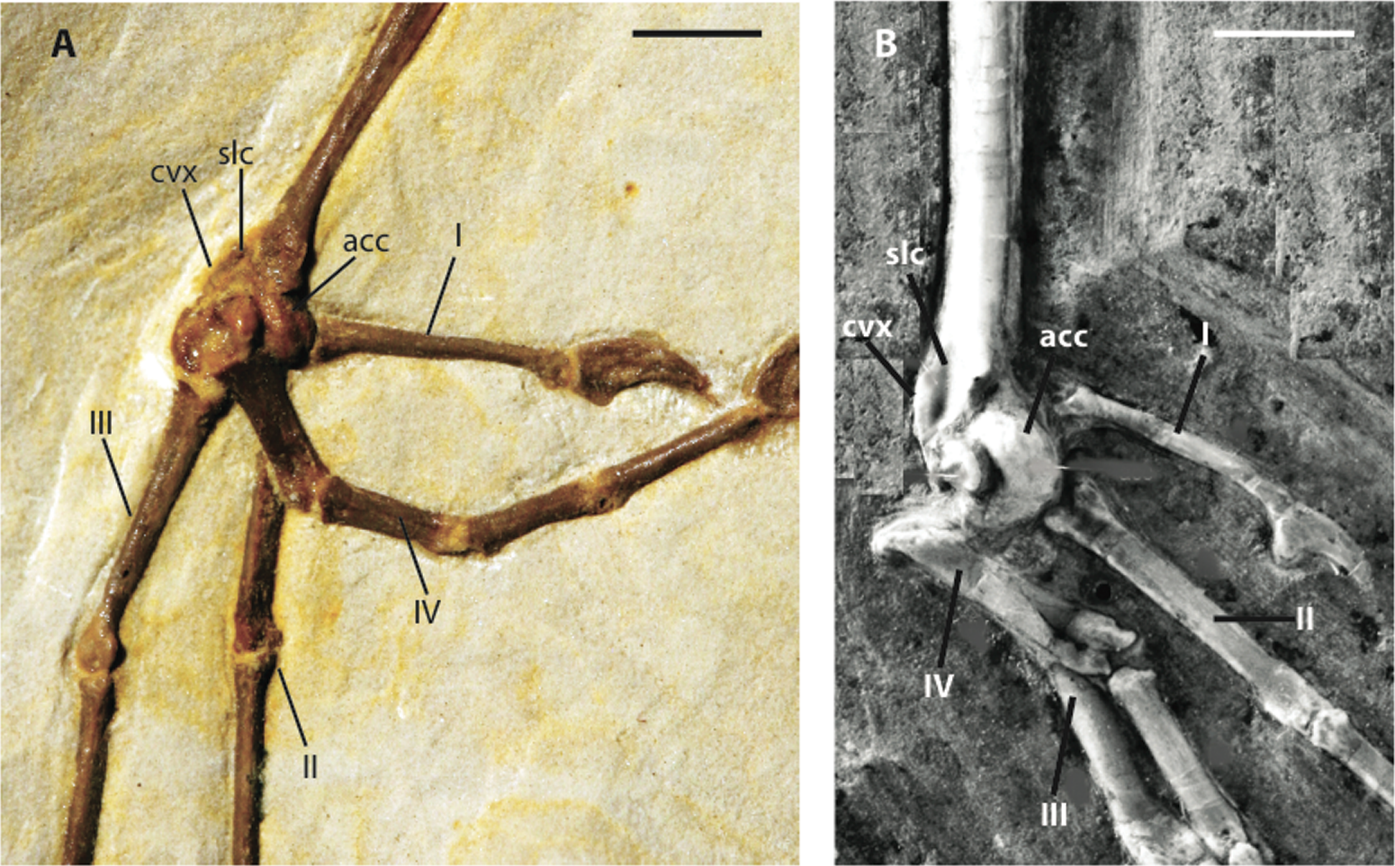
Distal left tarsometatarsi in lateroplantar view. (A) *Zygodactylus grandei*, FMNH PA 726; (B) *Zygodactylus luberonensis*. SMF Av 519. Anatomical Abbreviations: I, II, III, IV: digits one, two, three, and four, respectively; acc, trochlea accessoria; cvx, convexity on anterolateral margin; slc, sulcus on plantar surface of anterolateral convexity (a synapomorphy of *Zygodactylus luberonensis* and *Zygodactylus grandei*; B modified from Mayr, 2008). Scale bar equals 5 mm.

**Table 2:** Ratios of measurements for European and North American zygodactylids.

Taxon	H:U	H:C	H:F	H:TM	U:TT	U:TM	C:TM	TT:TM
*E. americanus* USNM 299821	0.88	1.91	–	–	–	–	–	–
*E. americanus* WDCCGR 014	0.94	1.95	0.87	0.79	0.56	0.83	0.40	1.40
*Z. grandei* FMNH PA 726	0.96	2.20	1.23	0.87	0.57	0.92	0.40	1.57
*P. danielsi* SMF ME 2522	0.88	2.13	1.0	0.89	0.72	1.02	0.42	1.43
*P. major* SMF ME 1758	0.92	2.24	1.15	1.03	0.79	1.11	0.46	1.41
*P. ballmanni* SMF ME 2108	0.88	2.23	1.0	0.84	0.72	0.95	0.38	1.33
*Z. luberonensis* SMF Av 519	0.95	2.0	0.87	0.70	0.52	0.70	0.35	1.42

**Notes:**

–, denotes missing data.

C, carpometacarpus; F, femur; H, humerus; TM, tarsometatarsus; TT, tibiotarsus; U, ulna.

**Table 3:** Measurements of selected zygodactylid specimens.

Taxon	H	U	C	F	TT	TM
*Z. grandei* FMNH PA 726	18.6	19.3	8.5	15.1	33.6	21.4
*E. americanus* FMNH PA 770	–	–	–	16.9	30.4	20.9
*E. americanus* USNM 299821	16.8	19.0	8.8	–	–	–
*E. americanus* WDC-CGR-014	17.2	18.2	8.8	19.7	30.6	21.8
*Z. luberonensis* SMF Av 519	17.2	18.1	8.6	19.5	34.7	24.5
*P. danielsi* SMF ME 2522	16.5	18.3	8.2	16.5	27.4	19.6
*P. major* SMF ME 1758	28.4	31.1	12	24.6	39.0	28.0
*P. ballmanni* SMF ME 2108	21.0	22.9	9.0	20.8	33.0	24.6
*P. eunjooae* HLMD-Me 10206	–	–	–	17.5	29.8	21.0
*P. quintus* SMF ME 11091	19.8	20.7	9.5	–	32.5	23.0
*P. longibrachium* SMF ME 11171	19.6	21.4	9.6	–	29.5	19.0

**Notes:**

All measurements are in millimeter and represent the maximum linear length of the element along the longitudinal axis.

–, missing data.

H, humerus; U, ulna; C, carpometacarpus; F, femur; TT, tibiotarsus; TM, tarsometatarsus.

**Table 4:** Measurements of Green River zygodactylids (in mm; left/right).

Taxon	CL	RL	NW	CL	HL	UL	CmL	FL	TL	TmL
*Z. grandei* FMNH PA 726	21.1	13.8	∼9.0	13.7/–	18.3/18.6	19.3/–	∼8.5	15.1/–	33.0/33.6	21.4/20.2
*Z. grandei* UWGM 40705	–	–	–	–	∼18.9	18.1	7.7	–	–	–
*E. americanus* WDC-CGR-014	–	–	–	13.8/13.1	–/∼17.2	∼18.2/–	8.8/–	19.7/–	–/30.6	21.7/21.8
*E. americanus* USNM 299821	∼19	∼13	∼8	–	16.8/16.8	∼19.0/∼19.1	–/8.8	–	–	–
*E. americanus* FMNH PA 770	–	–	–	–	–	–	–	16.9/–	30.4/30.1	20.9/20.5
Zygodactylidae gen. et. sp. indet.UWGM 21421	–	–	–	–	–	–	–	–/∼12.0	–/21.5	–/13.5
Zygodactylidae gen. et. sp. indet..FMNH PA 757	18.9	13.3	7.1	–	–	–	–	–	–	–
Zygodactylidae gen. et. sp. indet..UWGM 40363	∼17.6	12.9	6.3	–	–	–	–	–	–	–

**Notes:**

Rostrum length is measured from the tip of the premaxilla to just anterior to the frontal. Narial width is measured from the furthest anterior point of the narial opening to the furthest posterior point of the narial opening. Other lengths are taken from points of greatest distance. Measurements from WDC-CGR-014 and USNM 299821 are from Weidig (2010).

–, missing data.

CL, cranial length; RL, rostrum length; NW, narial width; CL, coracoidal length; HL, humeral length; UL, ulnar length; CmL, carpometacarpal length; FL, femoral length; TL, tibiotarsal length; TmL, tarsometatarsal length.

**Table 5:** Dimensions of pedal phalanges from known Green River and European zygodactylids for comparison.

Digit	*E. americanus* WDC-CGR-014	*E. americanus* FMNH PA 770	*Z. grandei* FMNH PA 726	*Z. luberonensis* SMF Av 519	*P. danielsi* SMF ME 2522	Zygodactylidae UWGM 21421
I:1	5.4/∼5.6	4.7/4.1	4.6/4.3	4.6	4	–
I:2	2.5/∼2.0	2.3/2.1	2.6/2.7	2.1	2.4	–
II:1	–/5.9	6.1/6.0	–/5.2	6.5	4.8	–
II:2	5.1/5.2	4.4/4.7	4.7/4.6	4.6	4.3	2.9
II:3	2.9/∼2.2	2.7/2.7	3.0/3.0	2.7	2.4	1.9
III:1	∼6.0/∼4.6	5.5/5.8	6.0/5.8	7.1	5.7	2.9
III:2	5.7/5.7	5.7/5.7	5.0/5.2	6.5	4.9	∼2.6
III:3	4.7/4.8	4.4/4.6	4.2/4.4	5.4	4.3	∼2.3
III:4	∼2.7/3.3	3.0/3.0	3.8/3.9	3.1	2.6	
IV:1	–/2.7	∼4.5/4.0	2.7/2.8	5.2	2.9	–
IV:2	2.8/2.9	2.8/2.5	2.7/2.5	4.0	2.9	–
IV:3	2.6/2.6	2.1/2.3	2.7/2.5	3.7	2.5	–
IV:4	2.6/2.6	3.0/2.8	3.3/3.4	3.1	2.5	–
IV:5	–/–	2.3/2.4	2.5/2.8	2.6	2	–

**Note:**

UWGM 21421 is a zygodactylid specimen of indeterminate genus and species. Measurements are in mm, L/R. Measurements for WDC-CGR-014 and SMF-2522 are from Weidig (2010) and Mayr (1998), respectively.

### Holotype specimen

FMNH PA 726, a partial, articulated skeleton comprising a complete skull and postcranial skeleton. Most of the right forelimb is absent, and much of the synsacrum is broken and crushed, obscuring morphology in those regions.

### Referred specimen

**Figure 10:**
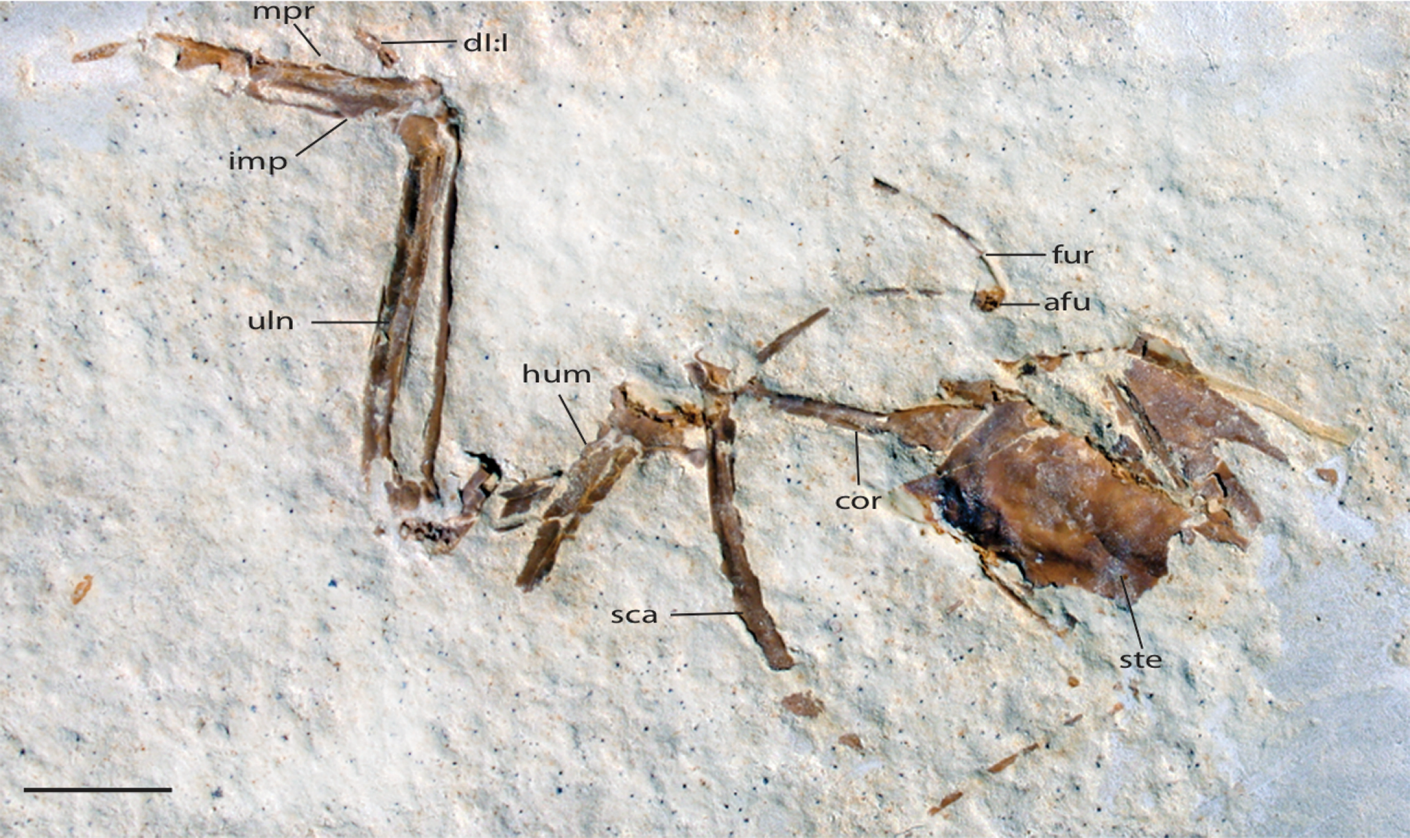
UWGM 40705, slab A, referred specimen of *Zygodactylus grandei*. Right forelimb, sternum and pectoral elements. Anatomical Abbreviations: afu, apophysis furculae; cor, coracoid; dI:I, digit one, phalanx one; fur, furcula; uln, ulna; hum, humerus; imp, intermetacarpal process; mpr, metacarpal process; sca, scapula; ste, sternum. Scale bar equals 1 cm.

**Figure 11:**
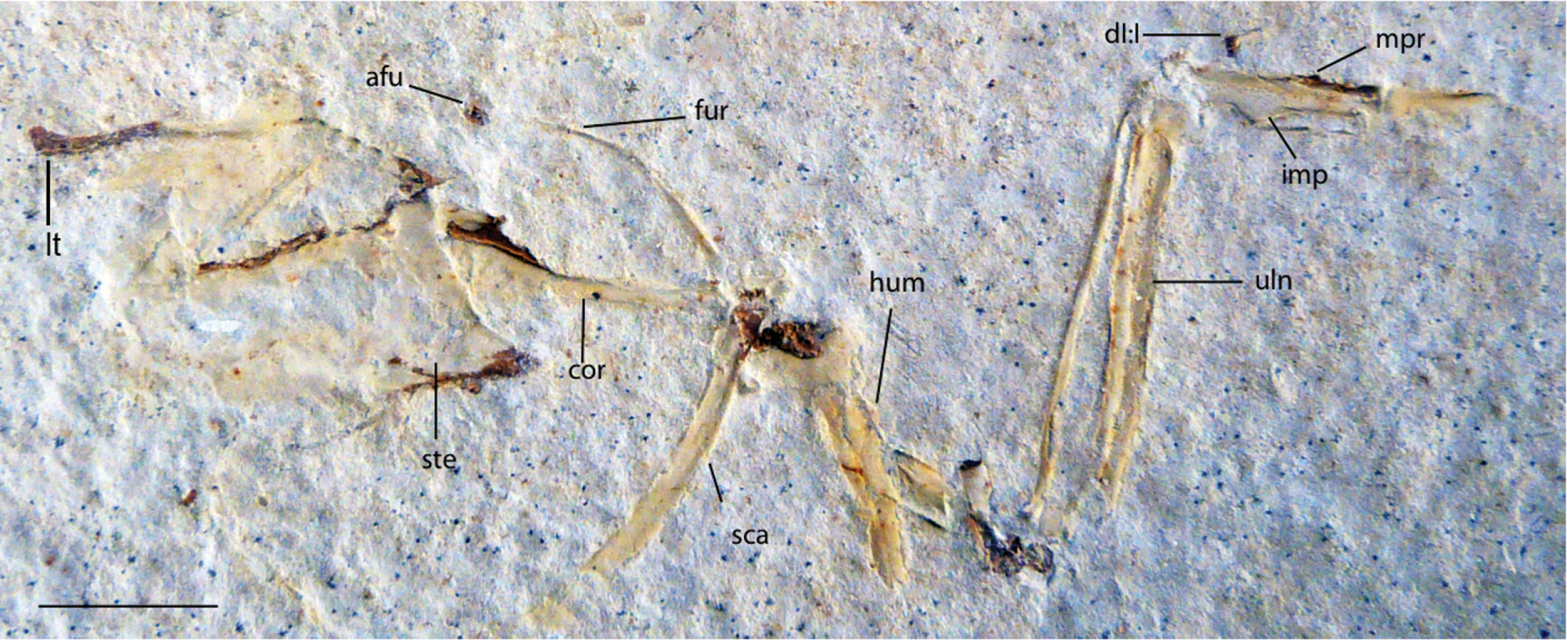
UWGM 40705, slab B, referred specimen of *Zygodactylus grandei*. Right forelimb, sternum and pectoral elements. Anatomical Abbreviations: afu, apophysis furculae; cor, coracoid; dI:I, digit one, phalanx one; fur, furcula; lt, lateral trabecula of sternum; uln, ulna; hum, humerus; imp, intermetacarpal process; mpr, metacarpal process; sca, scapula; ste, sternum. Scale bar equals 1 cm.

UWGM 40705, a partially articulated pectoral girdle and limb (Figs. 10 and 11) from Locality J of Grande (2013).

### Etymology

The species name “grandei” recognizes the extensive insight into the fauna of the Green River Formation gained by more than 30 years of research by Field Museum Curator Dr. Lance Grande.

### Locality and horizon

The holotype and referred specimens were collected from the Thompson Ranch Quarry near Kemmerer in Lincoln County, Wyoming, USA (“Locality J” of Grande, [2013]), part of the early Eocene FBM of the Green River Formation. Radiometric dating (40Ar/39Ar) of an overlying tuff indicates an age of approximately 52 Ma for the fossil-bearing horizon (Smith, Carroll & Singer, 2008; Smith et al., 2010).

### Diagnosis

*Zygodactylus grandei* sp. nov. is unique among taxa referred to Zygodactylidae in having a femur which is shorter than the humerus (Appendix I, character 21). Both the holotype and referred specimen possess a digit III ungual that is more than 25% the length of the sum of the three proximal phalanges (Appendix I, character 50; Table 5). These characters can be assessed in most described Zygodactylidae, save the fragmentary *P. minutus*, *Z. ignotus*, and *Z. grivensis*.

*Zygodactylus grandei* sp. nov. differs from *Z. luberonensis* in possessing a shorter tarsometatarsus (Table 3) and differs from *Z. grivensis* in possessing a shorter accessory trochlea (see Mayr, 2008, figure 1G). *Z. grandei* is more similar to *Z. luberonensis* both in the somewhat shorter accessory trochlea and in the presence of a marked sulcus on the plantar surface of the convexity on the proximal end of metatarsal trochlea IV (Mayr, 2008). A stouter coracoid with a more expanded sternal margin distinguishes *Z. grandei* from *Z. luberonensis* (Fig. 8). As in *Primozygodactylus, Z. grandei* possesses a small medial flange on the sternal end of the coracoid (not present in *Z. luberonensis*). This feature appears to be variably present in passeriforms and piciforms. Unfortunately, the coracoid in the paratype of *E. americanus* is poorly preserved, and no coracoid is exposed in the *E. americanus* holotype specimen. *Z. grandei* is further distinguished from the holotype and paratype of *E. americanus* by the presence of a protuberance (“dentiform process”) on the mid-shaft of the dorsal margin of metacarpal II (Weidig, 2010; Appendix I, character 28) and lateral trabeculae of the sternum that do not extend posterior to the medial trabeculae (Appendix I, character 17). In *P. minutus* the lengths of metacarpi II and III are nearly equal, whereas metacarpal III is longer in *Z. grandei* (Appendix I, character 32).

### Remarks

UWGM 40705, here referred to *Z. grandei*, was tentatively assigned to *E. americanus* by Weidig (2010). This specimen is strikingly similar in preserved morphology, size, and proportions with both the holotype and referred specimen of *E. americanus* and the holotype of *Z. grandei.* However, it shares with the holotype specimen of *Z. grandei* (FMNH PA 726) to the exclusion of *E. americanus*, a mid-shaft protuberance on the anterior margin of metacarpal II (Appendix I, character 28; “processus dentiformis,” Mayr, 2004). As noted above a dentiform process is absent in the holotype (USNM 299821) and paratype (WDC-CGR-014) of *E. americanus*, as well as in *Primozygodactylus*.

### Description

#### Skull

**Figure 12:**
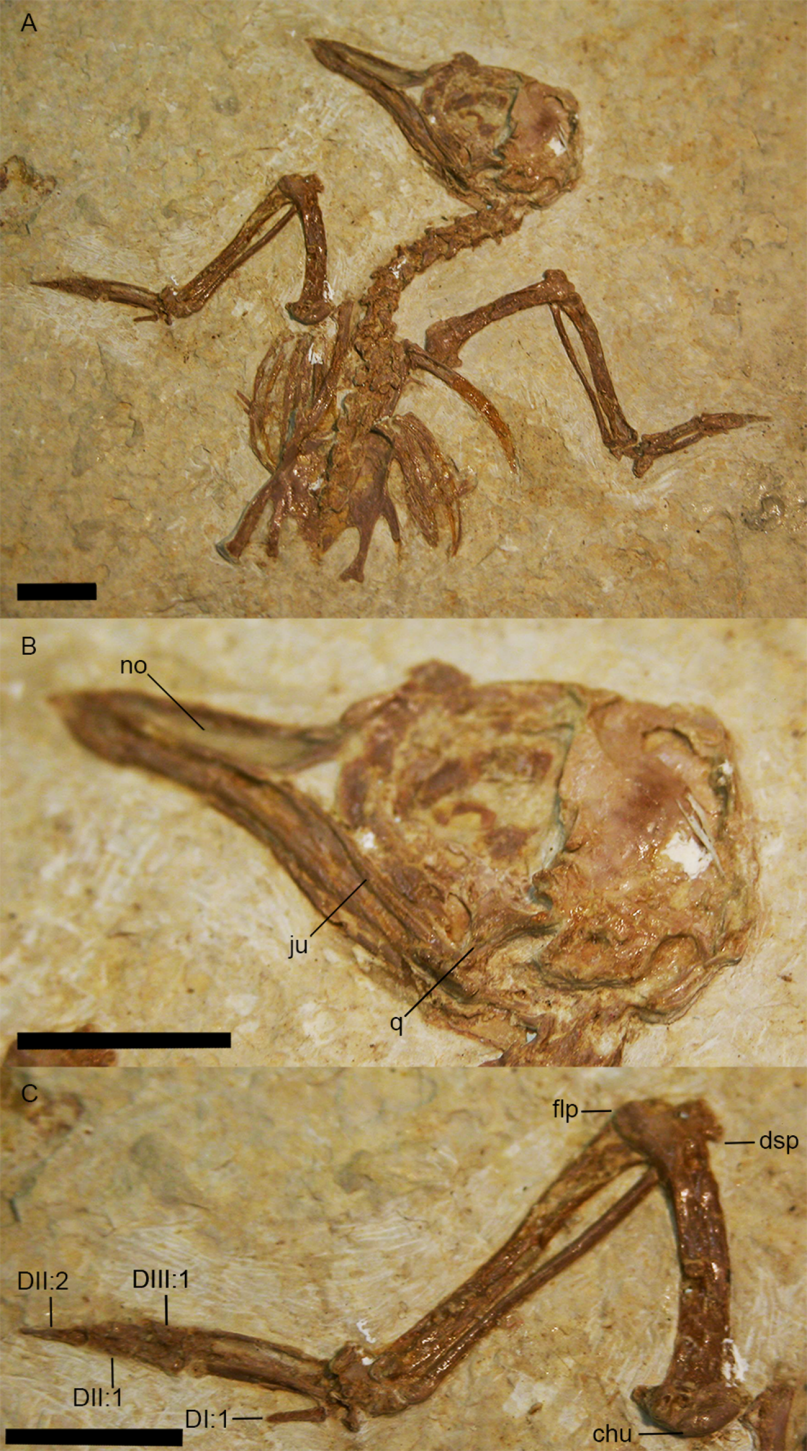
USNM 299821, holotype specimen of *Eozygodactylus americanus* (scale bar equals 1 cm).

The tip of the rostrum is slightly decurved (Figs. 2 and 3C). As in other species of Zygodactylidae (e.g., the holotype of *E. americanus*; Fig. 12), the narial openings are elongate, approaching three quarters of the overall rostrum length. The posterior and anterior-most portions of the narial openings are slightly fractured, which may slightly distort their shape. Within Aves, elongate, subrectangular narial openings also are seen in Columbiformes and some Passeriformes. The antorbital fenestrae are adjacent to the posterior-most edge of the narial openings with the narial bar nearly vertical in orientation. This condition contrasts with the substantial overlap of fenestrae and narial openings, and strongly angled narial bar seen in some parts of Coracii (e.g., *Coracias garrulus*). The specimen appears to lack an ossified nasal septum, as was noted previously in *Primozygodactylus*, Passeriformes and some Piciformes (namely members of Picidae; e.g., Mayr, 2009), and in contrast to the ossified nasal septae seen in Coraciiformes and other members of Piciformes (specifically Galbulidae and Bucconidae; Clarke et al., 2009). Thin, barely-visible segments of bone within the exposed narial opening may represent portions of vomer, commonly visible in this region.

Scleral ossicles are preserved in the orbit, although many are missing or shifted out of their original life position (Fig. 3C). Other ossicles are crushed, though the margins of five are distinctly visible. The relative ossicle sizes and shapes appear similar to those of ?*P. ballmanni* (HLMD-Me 15396, see figure 21 of Mayr, 1998). Overlying the dorsal edge of the posterior mandible is a thin and ventrally bowed jugal, which is obscured just anterior to the quadrate. The quadrate is three-dimensionally preserved. The orbital process is relatively short in comparison to the condition in extant Passeriformes, though unfortunately no quadrates of other zygodactylids are preserved. The dorsal border of the frontal, braincase, and portions of the quadrate and lacrimal/ectethmoid complex are severely crushed (Fig. 3C). However, a minute postorbital process is visible (similar in size and appearance to the same process in some passeriforms, e.g., *Tyrannus forficatus*).

#### Mandible

The mandible is exposed in left lateral view (Fig. 3C). The mandibular symphysis is short, and the posterior portion of the mandible is angles ventrally. The mandibular ramus curves ventrally from approximately its midpoint but appears slightly upturned where it terminates with an abbreviated retroarticular process. An elongate depression is developed on the posterolateral mandible, though it is unclear whether this is a morphological feature or the result of crushing and distortion. What may be a posterior mandibular fenestra (or possible separation due to breakage) is present slightly anterior to the lateral mandibular process; however, the paratype of *E. americanus* lacks this fenestra. Due to the possibility of breakage of this feature, it is coded as “?” in Appendix I.

#### Vertebral column

Thirteen individual cervical vertebrae and 21 total presacral vertebrae are visible (Figs. 3C, 4 and 5). There is no indication of a notarium. Crushing obscures fine morphological features in the anterior cervicals. The synsacrum is distorted and sacral number cannot be assessed. Only two anterior caudal vertebrae are exposed and the pygostyle is not preserved.

#### Pectoral girdle

The sternum is visible in dorsal view but is partially covered by ribs and matrix (Fig. 4). Posterior segments of the medial trabeculae are preserved as impressions, though the lateral trabeculae are intact. As in other zygodactylid specimens such as holotype of *E. americanus*, and specimens of *Primozygodactylus* (i.e., HLMD-Me 15396, HLMD-Me 10206, and WN 89609; see figure 24 of Mayr, 1998), the sternum is broad, and exhibits four deep caudal incisures. The lateral and medial trabeculae are approximately equal in posterior extent. This condition is also seen in the referred specimen of *Z. grandei* sp. nov. (UWGM 40705; Fig. 11). By contrast, the lateral trabeculae extend farther posteriorly than the medial trabeculae in the holotype specimen of *E. americanus*. The posterior tips of the trabeculae exhibit slight mediolateral expansion, but not as extensive as in *E. americanus*. A relatively broad, non-bifid, external rostral spine is present on the anterior edge of the sternum (Fig. 4). By contrast, the external rostral spine was reconstructed as comparatively narrow in primozygodactylids by Mayr (1998). In extant Passeriformes (Manegold, 2008) as well as Piciformes, the external rostral spine is typically bifid.

The left coracoid is visible in dorsal view (Fig. 4). It is not as slender as that of *Z. luberonensis* and has a relatively small acrocoracoid process that is partly obscured. In contrast with Piciformes, Trogoniformes, and some Coracii, no sternal notch is present. In contrast to *Z. luberonensis*, and in common with all described coracoids of *Primozygodactylus* (Mayr, 1998, 2008, 2009), a flange is located on the medial margin of the coracoid of *Z. grandei* (Fig. 8).

The scapula (Fig. 4) is relatively short and moderately recurved with a tapering distal tip and moderately well-developed acromion process similar to that observed in the holotype specimen of *P. danielsi* (SMF-ME 2522; see figure 22 of Mayr, 1998) and the holotype of *E. americanus*. The acromion process is not bifurcated as it is in many extant passeriforms (save Eurylaimidae and Cotingidae; Olson, 1971), piciforms and psittaciforms.

A relatively thin bone, which is adjacent to the right side of the left humerus, appears to be a part of the left furcular ramus (Fig. 7). Just medial to the right coracoid, a second narrow element appears to be a part of the right ramus. Although comparisons are limited for such a fragmentary element, preserved characteristics of the furcula are similar to that in the referred specimen of *Z. grandei* (UWGM 40705). Thin furcular rami are typical of Passeriformes, Piciformes and other zygodactylids. In the referred specimen of *Z. grandei*, the furcular apophysis is preserved. A blade-like apophysis, seen in passeriforms and some piciforms, as well as *P. danielsi* (Mayr, 1998), is absent and this area is broadly rounded. A rounded furcular apophysis is has also been reported in an unnamed species referred to *Primozygodactylus* (WN 89609; Mayr, 1998).

#### Pectoral limb

The right and left humeri of the specimen are exposed in posterior view (Figs. 4 and 7). Although the left humerus is comparatively well exposed, only the posterodorsal edge of the right humerus is visible. The bicipital crest is short. In contrast to the more well projected deltopectoral crest seen in *P. danielsi* (see figure 25 of Mayr, 1998), the less expanded deltopectoral crest of *Z. grandei* is similar to the condition observed in the holotypes of *E. americanus*, and *P. danielsi*, as well as *Primozygodactylus* sp. indet. (WN 88583A). The ventral tubercle is prominent, as in *E. americanus*, *Primoscens* sp. (WN 87558A), and the holotype of *P. danielsi* (Mayr, 1998).

The left humeral shaft is crushed, such that its midpoint width cannot reliably be assessed (Fig. 7). The curvature of the shaft appears to be less than that seen in the holotype of *E. americanus*, although the decreased curvature of *Z. grandei* may be an artifact of the aforementioned crushing. On the distal humerus, a well-projected dorsal supracondylar process is visible, which was suggested by Mayr (2008) to be a local synapomorphy of a clade containing *Zygodactylus* and Passeriformes (though this feature is present in Piciformes as well). The projection and size of the process is similar in appearance to that of WN 92747 (“Primoscenidae” indet.; see figure 25 of Mayr, 1998) and *Z. luberonensis* (Mayr, 2008) in that it is projected somewhat anterodorsally with a small notch separating it from the shaft. By contrast, it is somewhat more dorsally directed than in the holotype and paratype of *E. americanus* (Weidig, 2010). Crushing obscures detail on the relative development of the m. scapulotriceps and m. humerotriceps grooves (i.e., the tricipital sulci). The flexor process is well-projected and bulbous, similar in size and shape to that of WN 92747 (“Primoscenidae” indet., Mayr, 1998) and extant Passeriformes (e.g., *Turdus merula*).

The left ulna is comparatively well exposed in oblique dorsal view, and a moderately projected olecranon process is visible. The right ulna is not exposed, and the proximal end of the left is partially obscured by the humerus. The ulna is longer than the humerus, as in *Z. luberonensis, Pici, Primozygodactylus danielsi* (SMF-ME 2522), ?*P. ballmanni* (HLMD-Me 15396), *P. major* (SMF-Me 1758), and the holotype and paratype of *E. americanus* (USNM 299821 and WDC-CGR-014). Passeriformes, by contrast, exhibit wider variation with ulnae that are shorter than, approximately equal to, or longer than the humerus. As in the *E. americanus* paratype, the ulna is shorter than the tarsometatarsus, unlike the condition seen in *Primozygodactylus* in which the ulna is longer or subequal to the tarsometatarsus (Weidig, 2010). These proportions also contrast with the generally elongate ulnae but short tarsometatarsi seen in many extant passeriforms and piciforms.

The left carpometacarpus is exposed in dorsal view but is partially covered proximally by the ulna and radius. The right carpometacarpus is not visible. A large intermetacarpal process (Fig. 7) is shared with the referred specimen UWGM 40705, all previously described taxa within Zygodactylidae for which a carpometacarpus is preserved, as well as extant Passeriformes and Piciformes. In contrast to all extant Passeriformes and some Piciformes, the intermetacarpal process is not fused to the third metacarpal, though it does contact this metacarpal. A carpometacarpal protuberance (“processus dentiformis” sensu Mayr, 2004) is present on the anterior surface of metacarpal II, as in Passeriformes, *Z. luberonensis* and *P. minutus*, though such a process is absent in *Primozygodactylus* and *E. americanus*. This feature is also found in the extinct sylphornithids (Mayr, 2004) and some kingfishers (Boles, 1997b).

Metacarpal III projects farther distally than metacarpal II (Fig. 7), a condition present in an array of avian taxa including most extant passeriforms (Livezey & Zusi, 2006, 2007; Manegold, 2008), *Z. luberonensis*, *Z. ignotus*, extant galbulids and the extinct sylphornithids (Mayr, 2004). This condition is absent in the three species of *Primozygodactylus* in which carpometacarpi are preserved (*P. ballmanni*, *P. danielsi,* and *P. major*; Mayr, 2004) and *P. minutus*. Metacarpals II and III are approximately equal in distal extent in these taxa. The distal carpometacarpus is poorly preserved in the holotype and paratype of *E. americanus*. However, in those specimens, metacarpal II appears to be slightly shorter than metacarpal III.

The manual phalanges are exposed on the left side (Fig. 7). The first digit has two phalanges and a small claw is present. Phalanx I:1 is comparable in size and appearance to the phalanges in the holotype and paratype of *E. americanus* and appears more elongate and slightly more gracile than that of *Primozygodactylus* (e.g., *P. danielsi*). Species of *Primozygodactylus*, including *P. eunjooae* and *P. danielsi*, also possess a manual digit I:2, and *Z. luberonensis* has a well-preserved phalanx I:2 that is virtually identical in size and appearance to that seen in *Z. grandei*. While non-preservation of such the delicate ungual of digit I cannot definitively speak to its absence, it is interesting to note that this ungual is present in both *Z. grandei* and *Z. luberonensis* but was not observed in specimens of *E. americanus* (e.g., USNM 299821, WDC-CGR-014) that show an apparently similar quality of preservation.

Phalanx II:2 is relatively narrow and shorter than II:1. In these morphologies, the new species resembles *P. danielsi* and *P. eunjooae* (Mayr, 2009). Passeriformes examined for this study have much more abbreviated and broad II:2. The proximally projected process on the anteroproximal tip of II:1, present in Piciformes (Mayr, 2004), is absent. Phalanx III:1 is narrow, and the flexor tubercle is inconspicuous, whereas the examined piciforms have a pronounced flexor tubercle on III:1. Phalanx II:1 is generally shorter and the flexor tubercle of phalanx III:1 is more well developed in Alcidinidae, Meropidae, Motmotidae, and Coraciidae.

#### Pelvic girdle

Portions of both the right and the left anterior iliac blades are visible in dorsal view with squared anterior margins (Fig. 5) similar to those of *P. danielsi* and *P. ballmanni*. A well-developed dorsolateral iliac crest is present. The posterior terminus of the ischium, visible on the left side, extends significantly beyond the terminus of this crest and is strongly angled ventrally. The left pubis is visibly rod-like and extends further posteriorly than the ischium.

#### Pelvic limb

**Figure 13:**
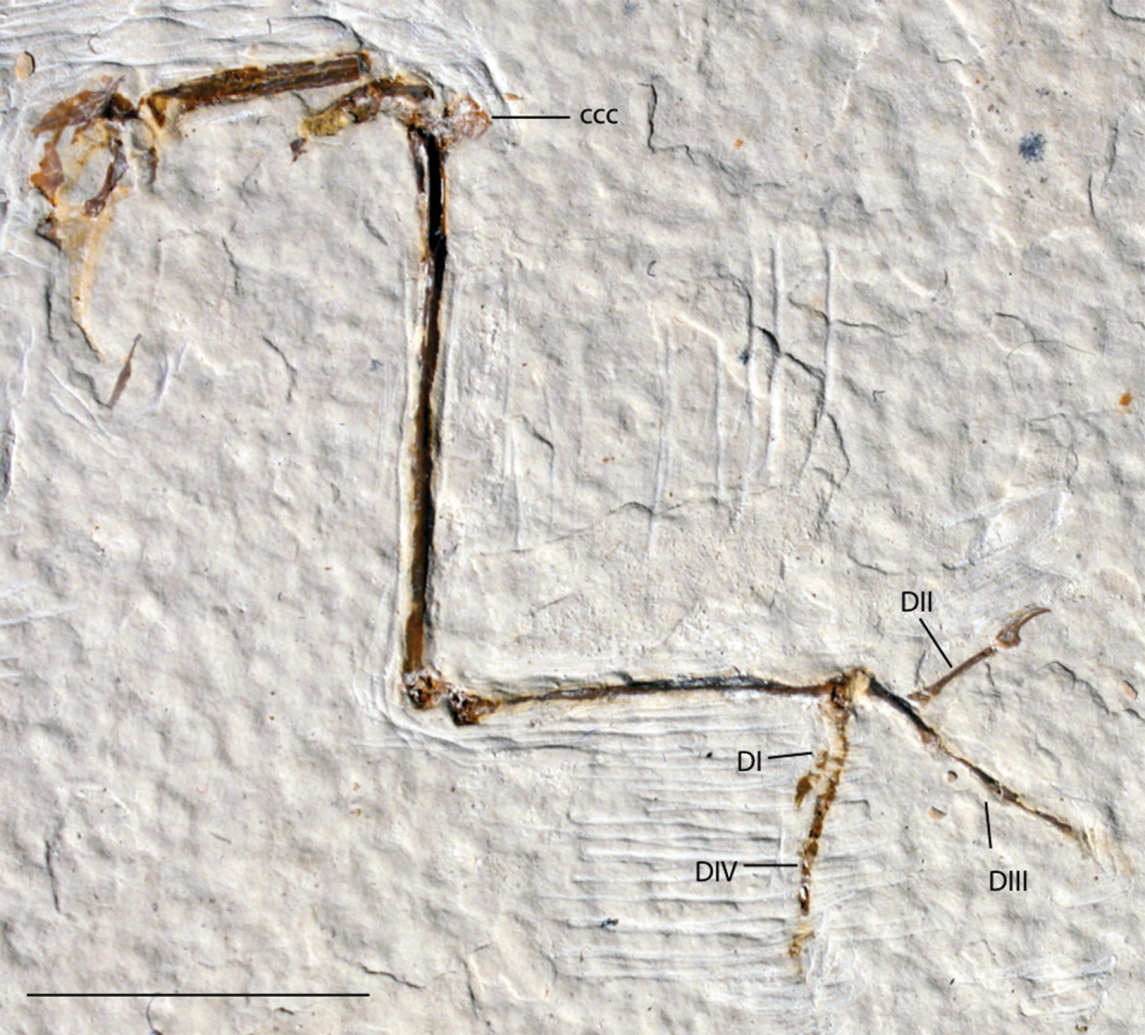
UWGM 21421, Zygodactylidae gen. et sp. indet. Note the well projected cranial cnemial crest and the retroversion of digits I and IV. Anatomical Abbreviations: ccc, cranial cnemial crest; DI, DII, DIII, DIV: digits one, two, three, and four, respectively. Scale bar equals 1 cm.

The femur is partially exposed. The proximal half of the right femur lies in articulation with the acetabulum, although the distal portion of element lies underneath other pelvic elements. The left tibiotarsus is exposed in lateral view and the right tibiotarsus is exposed in medial view (Fig. 6). The fibula lies in articulation with the proximal left tibiotarsus. The tibiotarsus is the longest hind limb element, as in *Primozygodactylus*, previously described species of *Zygodactylus*, and the *E. americanus* paratype specimen. The cranial cnemial crest, clearly visible on the right tibiotarsus, is pronounced and well projected anteriorly. This condition is also seen in *E. americanus, Z. luberonensis* and all specimens of *Primozygodactylus* in which the proximal tibiotarsus is exposed. However, it is weakly projected in comparison with the cranial cnemial crest in the leg of a small zygodactylid specimen (UWGM 21421), which was assigned to Zygodactylidae gen. et. sp. indet. by Weidig (2010; Fig. 13). The condyles project posteriorly relative to the tibiotarsal shaft in a conformation similar to that of Passeriformes. The cnemial crest is not hooked, in contrast to the condition in most Passeriformes, Piciformes and *Z. luberonensis*. The cnemial crest does not appear to be hooked in the paratype of *E. americanus*.

The left tarsometatarsus is exposed in lateral view and the right tarsometatarsus is exposed in medial view (Figs. 2 and 9A). The medial and lateral plantar crests are relatively distinct, and thus the tarsometatarsus would likely have had a similar cross-sectional shape to that of most extant passeriforms (i.e., subtriangular). The presence of accessory trochlea and the preservation of the fourth toe in a retroverted position on both the right and left foot, strongly support the interpretation that *Z. grandei* was indeed, zygodactyl. The tarsometatarsus is thin and elongate, as in Passeriformes, Piciformes, the paratype of *E. americanus*, other Zygodactylidae species (e.g., *Z. luberonensis*, *P. danielsi*, *P. ballmanni*, *P. major*), *Gracilitarsus mirabilis*, and an array of other avian taxa that are otherwise quite distinct from *Z. grandei*. However, relative to humeral and ulnar length, the tarsometatarsus is proportionally longer in *Z. grandei* than in species of *Primozygodactylus* and most species of Passeriformes examined for this study. The right proximal tarsometatarsus is partially visible in plantar view and shows at least one ossified hypotarsal canal. The medial hypotarsal crest is visible on both the left and right tarsometatarsus (Fig. 2), though details are difficult to ascertain due to crushing. The hypotarsus is large and well projected plantarly with a deep medial parahypotarsal fossa as in other *Primozygodactylus* and *Zygodactylus* specimens with preserved tarsometatarsi. The intercotylar eminence is relatively diminutive.

Metatarsal I is visible on the right foot and is relatively abbreviated (Fig. 9A). The trochlea of metatarsal III extends farthest distally, and metatarsal II extends farther distally than metatarsal IV. A small plantar ala is also developed on the trochlea of metatarsal IV. Well-preserved accessory trochleae on metatarsal IV are visible on both feet. They are extremely well projected plantarly, as in *Z. luberonensis*, though in contrast to described specimens of *Primozygodactylus*. However, in contrast with the paratype of *E. americanus*, the metatarsal III trochlea projects distal to the metatarsal II and IV trochleae. As mentioned above, *Z. grandei* exhibits a previously proposed apomorphy of *Zygodactylus* (Mayr, 2008), a distinct convexity on lateral tarsometatarsal margin just proximal to the metatarsal IV trochlea (Fig. 9A; Appendix I, character 39). It also exhibits a feature proposed by Mayr (2008) as an autapomorphy of *Z. luberonensis*, the presence of a marked sulcus on the plantar surface of the proximal end of trochlea metatarsal IV bordering the aforementioned lateral tarsometatarsal convexity (Mayr, 2008; Fig. 9; Appendix I, character 40). This character can no longer be considered an autapomorphy of *Z. luberonensis*, but is instead, an apomorphy with a known distribution restricted to *Zygodactylus*.

As in *Z. luberonensis* and *E. americanus*, the phalanges are more gracile, and the unguals apparently less recurved than those of *Primozygodactylus* (Fig. 9; see figure 2 of Mayr & Zelenkov, 2009). Digit I is relatively elongated with a slightly recurved ungual. The digits are longer and thinner than all described specimens of *Primozygodactylus* (Table 5), though they are similar in size and proportions to *E. americanus* and *Z. luberonensis* (see figure 2 of Mayr, 2008). As in all other Zygodactylidae, the unguals exhibit a pronounced vascular sulcus (Mayr, 1998, 2008, 2009). That the digit III ungual is more that 25% of the sum of the lengths of the more proximal phalanges (III:1–3) is proposed to represent an autapomorphy of *Z. grandei*. In other zygodactylids, *A. chloris*, and other examined passerines (e.g., *Tyrannus tyrannus, Thamnophilus caerulescens, C. bracnyrunchus, M. novahollandiae*) this ungual is <25% of the sum of the lengths of the more proximal phalanges.

***Eozygodactylus americanus*** Weidig, 2010
(Fig. 12)

**Type Specimens:** *Holotype specimen* USNM 299821, partially articulated skeleton lacking pelvis and hind limbs; *Paratype specimen* in Weidig (2010)-WDC-CGR-014, articulated postcranial skeleton (skull not preserved)

**Type Locality:** Tynsky Quarry (Locality H of Grande, [2013]), near Kemmerer, Lincoln County, Wyoming, USA, Type Horizon: Green River Formation, FBM.

**Figure 14:**
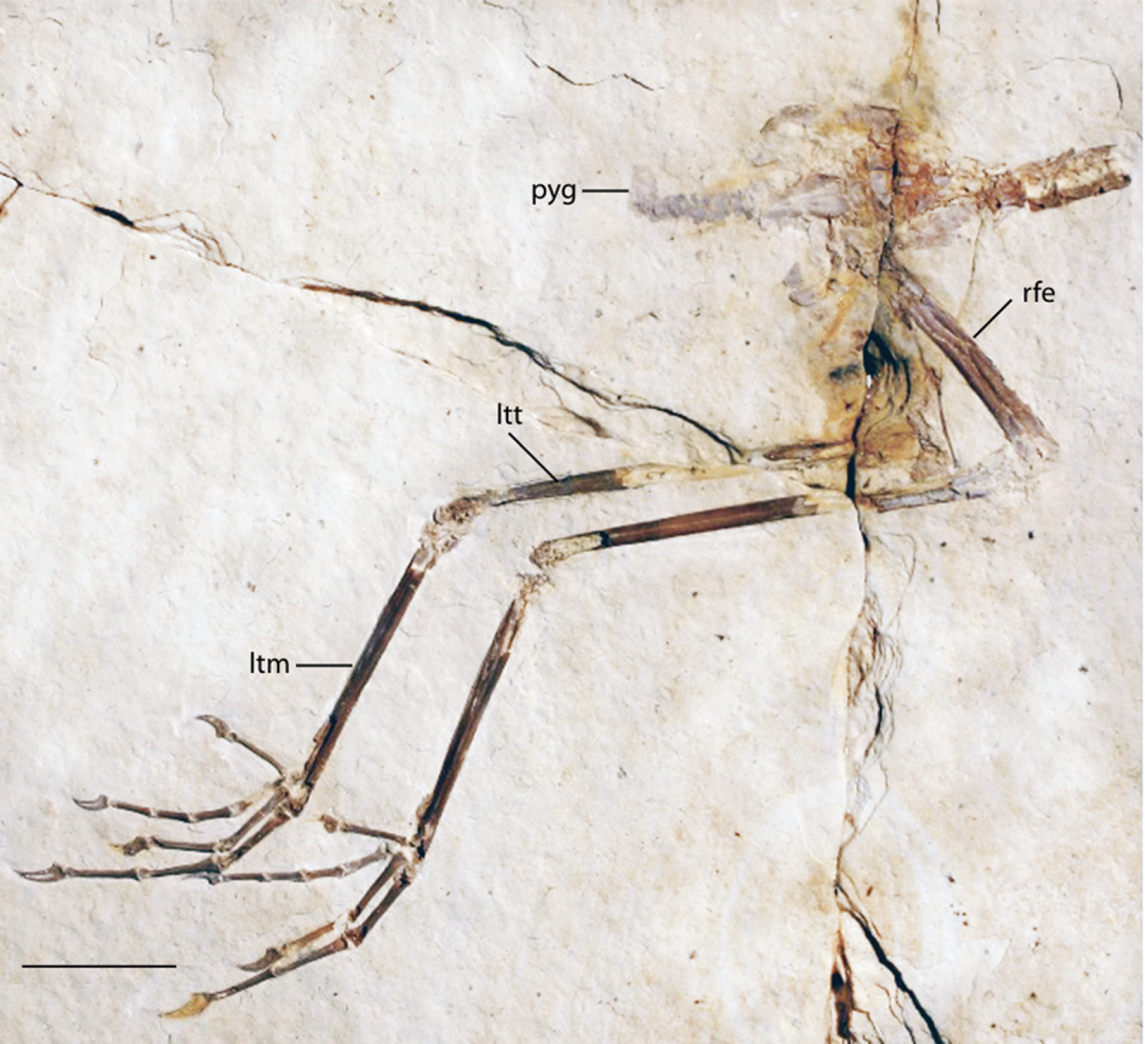
FMNH PA 770 slab A, tentatively referred to *Eozygodactylus americanus*. Exposed in right lateral view. Anatomical Abbreviations: ltm, left tarsometatarsus; ltt, left tibiotarsus; pyg, pygostyle; rfe, right femur. Scale bar equals 1 cm.

**Figure 15:**
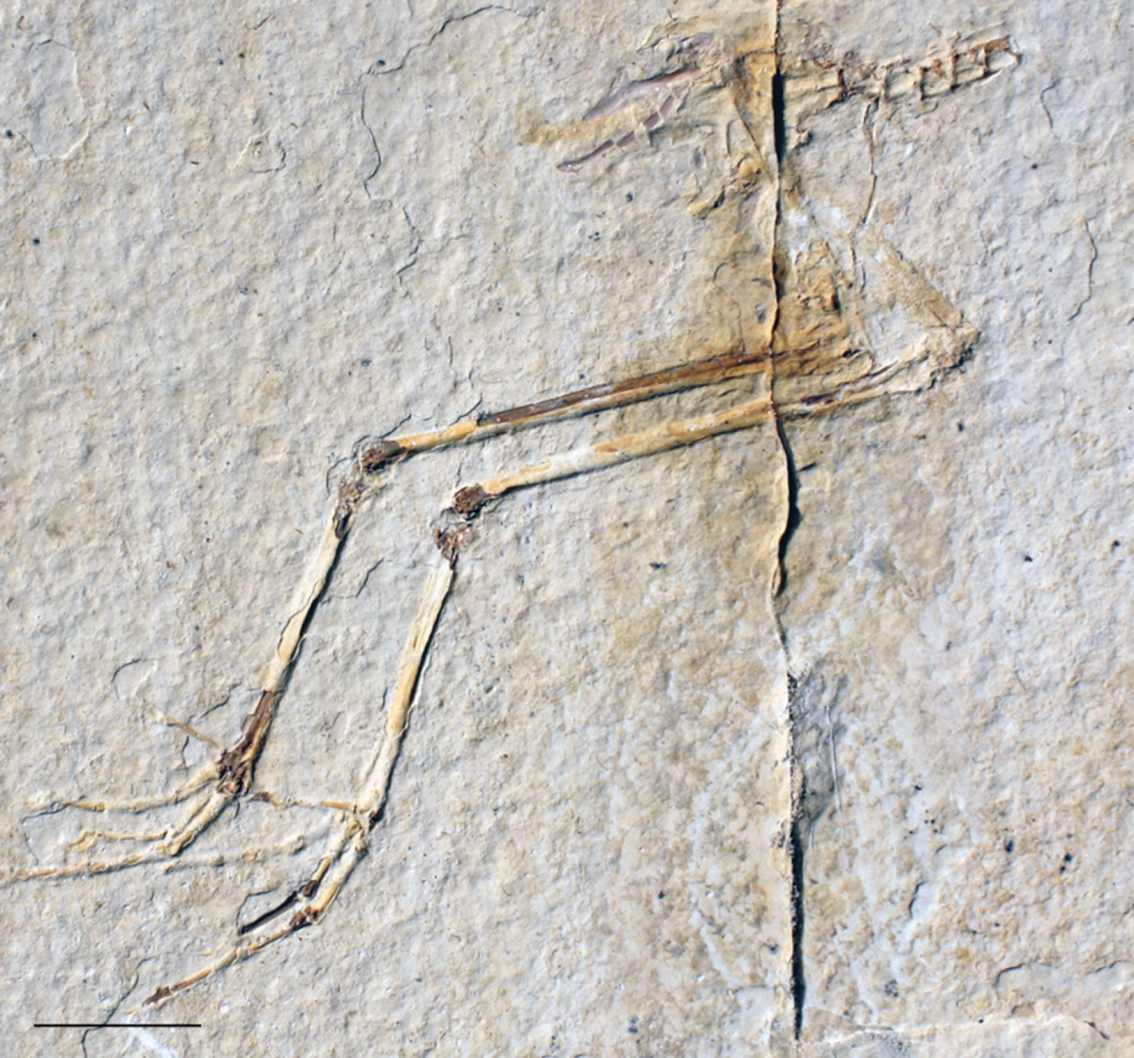
FMNH PA 770, slab B, tentatively referred to *Eozygodactylus americanus*. Exposed in right lateral view. Scale bar equals 1 cm.

**Newly Referred Specimen:** FMNH PA 770, Slab A and B, partial pelvic girdle and articulated pelvic limbs (Figs. 14 and 15), from Locality H of Grande (2013)

**Emended Diagnosis:** *Eozygodactylus americanus* was diagnosed by Weidig (2010) based on the following combination of characters: the presence of a humerus with a large dorsal supracondylar process (Appendix I, character 24); manual digit III:1 widened into a small tubercle (Appendix I, character 33); pelvis with an open obturator foramen (Appendix I, character 36). A pronounced dorsal supracondylar process is also present in *Z. luberonensis*, *Z. grandei*, and undescribed zygodactylid (WN 92747; Mayr, 1998). In contrast to *E. americanus*, the obturator foramen is closed in *Primozygodactylus*; however, that feature can only be assessed in *P. danielsi*, *P. ballmanni*, and *P. major* due to incomplete preservation in other specimens and an open obturator foramen is widespread within Aves (Livezey & Zusi, 2006, 2007). We note that the proportions of the holotype and the paratype specimens of *E. americanus* differ (Tables 3 and 4). The holotype has a well-preserved skull but the hind limbs were not preserved, whereas the paratype has no skull but has preserved hind limbs. More detailed examination of these two specimens and the recovery of more comparative material may reveal that the paratype and holotype actually represent different species. *E. americanus* can be distinguished from *Z. luberonensis*, *Z. grandei*, and *P. minutus* by the absence of a dentiform process on the carpometacarpus (Appendix I, character 28). The paratype of *E. americanus* can additionally be distinguished from *Z. grandei* by size-based differences; pedal ungual III:4 is less than 25% of the sum of the length of the proximal three phalanges of digit III. Further, the paratype of *E. americanus* does not share the foreshortened femur of *Z. grandei* (Tables 3 and 4).

### Description

FMNH PA 770 shares the following morphological features with the *E. americanus* paratype specimen (WDC-CGR-014): elongate toes and gracile unguals (Appendix I, character 48); almost identical measurements (Table 4); and an ungual on pedal digit III which is <25% the length of the sum of the lengths of the three more proximal phalanges of this digit (Appendix I, character 50). Slab A, which contains the majority of preserved elements of FMNH PA 770, comprises posterior thoracic vertebrae, free caudal vertebrae, a pygostyle, pelvic girdle and complete right and left hind limbs exposed in right lateral view. The tip of the right ischium is preserved as an impression. The texture of the bone at the tarsometatarsal and tibiotarsal epiphyses is unfinished (i.e., exposed trabeculae), suggesting that this specimen may represent a subadult individual. Slab B primarily consists of impressions of the opposite side of the specimen, with a few fragments of preserved bone, including the right ungual of digit III, portions of proximal phalanges II and III, and several fragments of the distal left tarsometatarsus. Unless otherwise stated, descriptions below focus on the substantially more complete slab A.

The preacetabular ilium is partially visible adjacent to the crushed synsacral vertebrae. Impressions of what are inferred to be postacetabular iliac blades are visible. The pygostyle is neither as enlarged nor disc-shaped as in piciforms (Appendix I, character 39) and is lacking the pronounced dorsal notch of trogoniforms. A pygostyle was not previously discernable in any described specimen of Zygodactylidae.

The femur is similar in width to *Z. grandei* (FMNH PA 726; Fig. 2). The tibiotarsus is thin and elongate, longer than the tibiotarsus of *Primozygodactylus*, though slightly shorter than *Z. grandei*, and almost identical to the tibiotarsal length of the paratype specimen of *E. americanus* (Table 3). Limb proportions in *Z. grandei* and *E. americanus* are somewhat different (see Table 2). Although details of the distal tarsometatarsi are not visible, the pedal phalanges are articulated and well preserved. An accessory trochlea is not visible. However, in both feet, digits II and III are exposed only in dorsal view, while digit IV on the right foot is preserved in palmar view, suggesting digit IV’s retroversion. The pedal phalanges are elongated and thin, as in the paratype of *E. americanus*, *Z. luberonensis*, and *Z. grandei*. By contrast, the phalanges of *Primozygodactylu*s are comparatively stout. The pedal unguals are short and only weakly recurved, as in *Z. grandei* and the paratype of *E. americanus*. The pedal unguals are larger and more recurved in *Primozygodactylus*.

**Figure 16:**
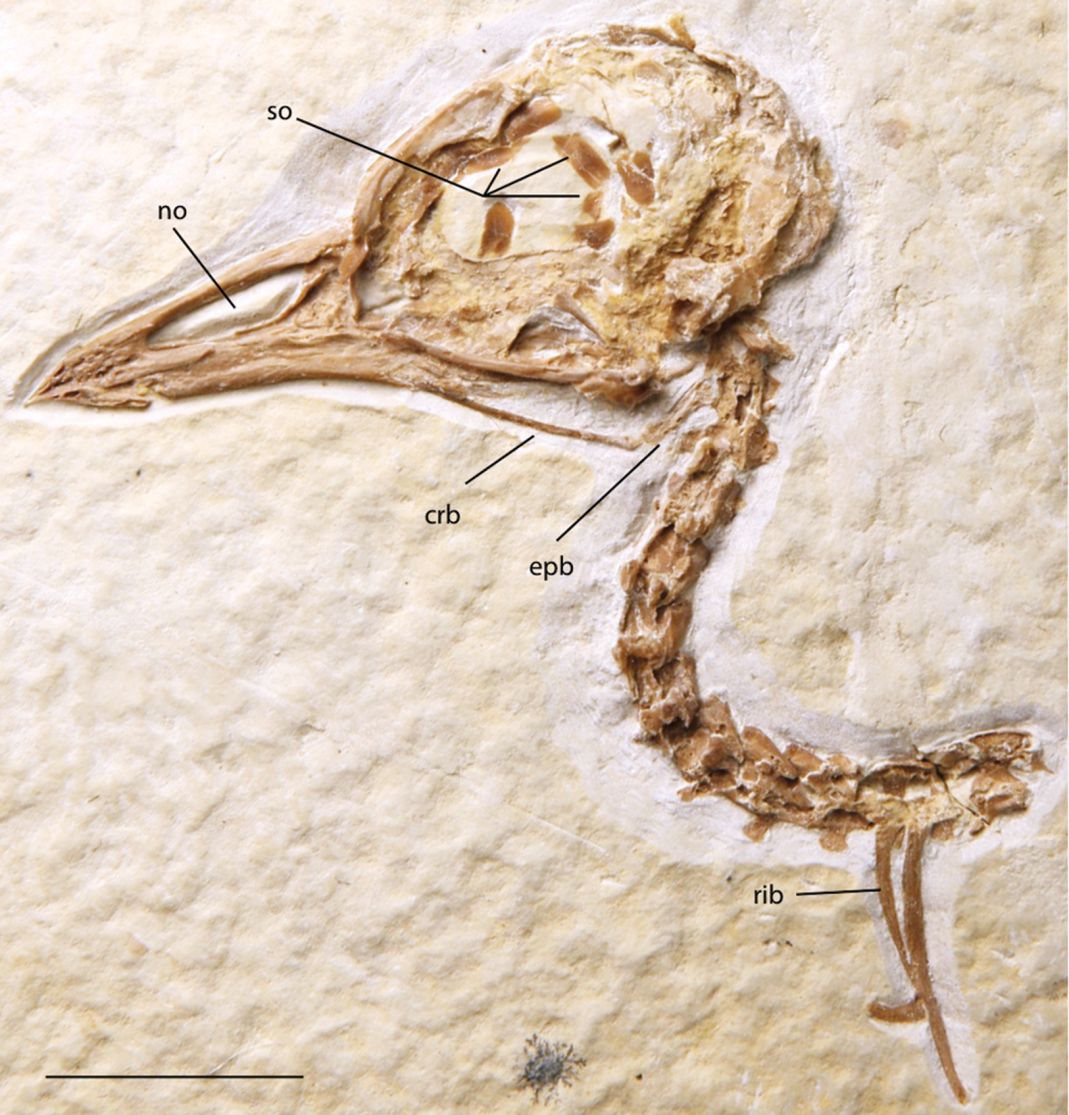
FMNH PA 757, referred to Zygodactylidae gen. et sp. indet. Anatomical Abbreviations: crb, ceratobranchial; epb, epibranchial; no, narial opening; so, scleral ossicles. Scale bar equals 1 cm.

**Figure 17:**
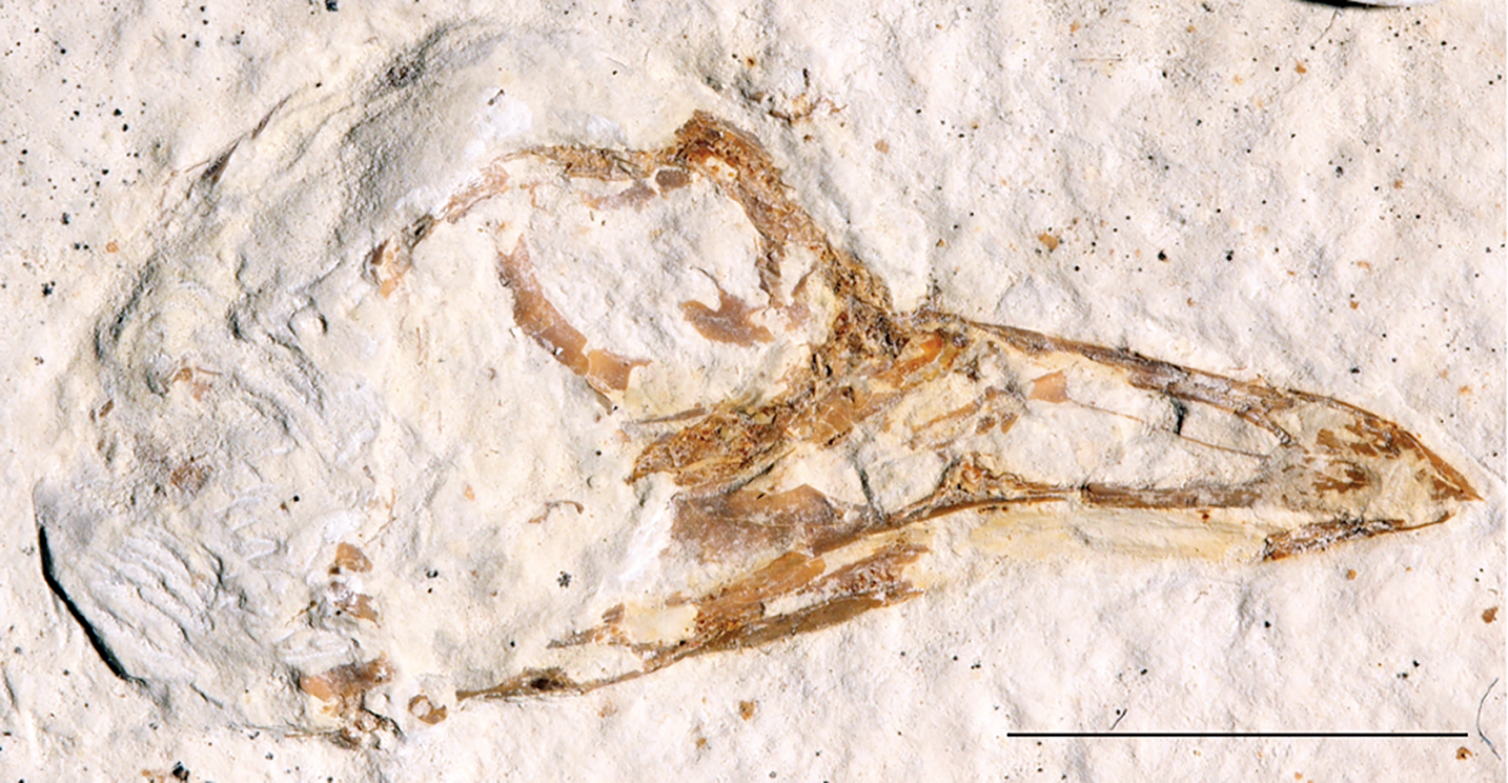
UWGM 40363, referred to Zygodactylidae gen. et sp. indet. Anatomical Abbreviations: crb, ceratobranchial; epb, epibranchial; no, narial opening; so, scleral ossicles. Scale bar equals 1 cm.

**Zygodactylidae gen. et sp. indet.**
(Figs. 3A, 3B, 16 and 17)

### Referred specimens

FMNH PA 757 (Figs. 3A and 16; FBM of the Green River Formation; Locality H of Grande (2013), skull and anterior cervical vertebrae preserved in left lateral view; UWGM 40363 (Figs. 3B and 17; FBM of the Green River Formation; Locality J of Grande [2013]), isolated skull in right lateral view.

### Remarks

Of the five FBM specimens treated herein, two (FMNH PA 757, UWGM 40363) are referred to Zygodactylidae indeterminate. They cannot with confidence be referred to either of the two zygodactylid species from the FBM, because the only preserved characters on these specimens are cranial. Known cranial elements of *Z. grandei* and *E. americanus* are indistinguishable on the basis of size or morphology.

### Basis for referral

FMNH PA 757 and UWGM 40363 are referable to Zygodactylidae based on the presence of a subrectangular narial opening (Appendix I, character 1), a narial opening greater than 50% of the length of the rostrum (Appendix I, character 2), and a ventrally bowed jugal (Appendix I, character 3). Further similarities shared by these specimens with Zygodactylidae include an absence of an internarial septum, the presence of thin nasal bars, and a recurved and pitted beak tip. Cranium length, rostrum length, and length of the narial opening in both specimens are nearly identical to the holotype specimens of *E. americanus* and *Z. grandei* (Table 4).

### Description of newly referred material

FMNH PA 757 is a skull preserved in left lateral view with several anterior cervical vertebrae preserved in articulation (Fig. 16). In all comparable morphologies and size UWGM 40363 is morphologically indistinguishable from FMNH PA 757 and other cranial material from the Fossil Butte zygodactylids, *Z. grandei* and *E. americanus*. Below, we focus on the better-preserved FMNH PA 757.

The rostrum is uncrushed anteriorly and shows numerous neurovascular pits and canals at its tip. Similar pits and canals are also visible in *Z. grandei* and UWGM 40363 (Fig. 3) but are poorly preserved. As in *Z. grandei* and *E. americanus*, the premaxillae are straight with a very slight downward curve near the tip of the rostrum. The narial opening of FMNH PA 757 is elongated; however, more triangular than in other, less well-preserved specimens attributed to the clade. The narial opening encompasses more than half of the total rostrum length. The narial bar is slightly angled with the anterior tip of the antorbital fenestra nearly even with, but just slightly anterior to, the posterior-most edge of the narial opening. The specimen shares with the holotype of *E. americanus* and the holotype of *Z. grandei* an elongated narial opening that lacks an ossified nasal septum. The nasofrontal hinge is well demarcated. An interorbital septum is largely absent, and the ossification of the mesethmoid appears to be limited. Its preserved shape is nearly identical to that in the holotype of *Z. grandei.*

Scleral ossicles are visible in the orbit, though many are shifted out of articulation. The margins of six are distinctly visible, and the relative ossicle sizes and shapes are not markedly different from those of *Z. grandei*. Overlying the dorsal edge of the posterior mandible is a thin and ventrally bowed jugal. The outline of the quadrate is visible in rough articulation with the jugal. Its orbital process, like that of *Z. grandei*, is more abbreviated than in most Passeriformes. The preservation of the orbital process in other zygodactylids is too poor for comparison. The braincase and anterior portions of the skull are crushed. Interestingly, this specimen preserves some of the hyoid apparatus. The preserved parts of the left ceratobranchial and elongate epibranchial are not helpful for comparisons with other zygodactylids, for which no hyoid material is described.

### Phylogenetic results

**Figure 18:**
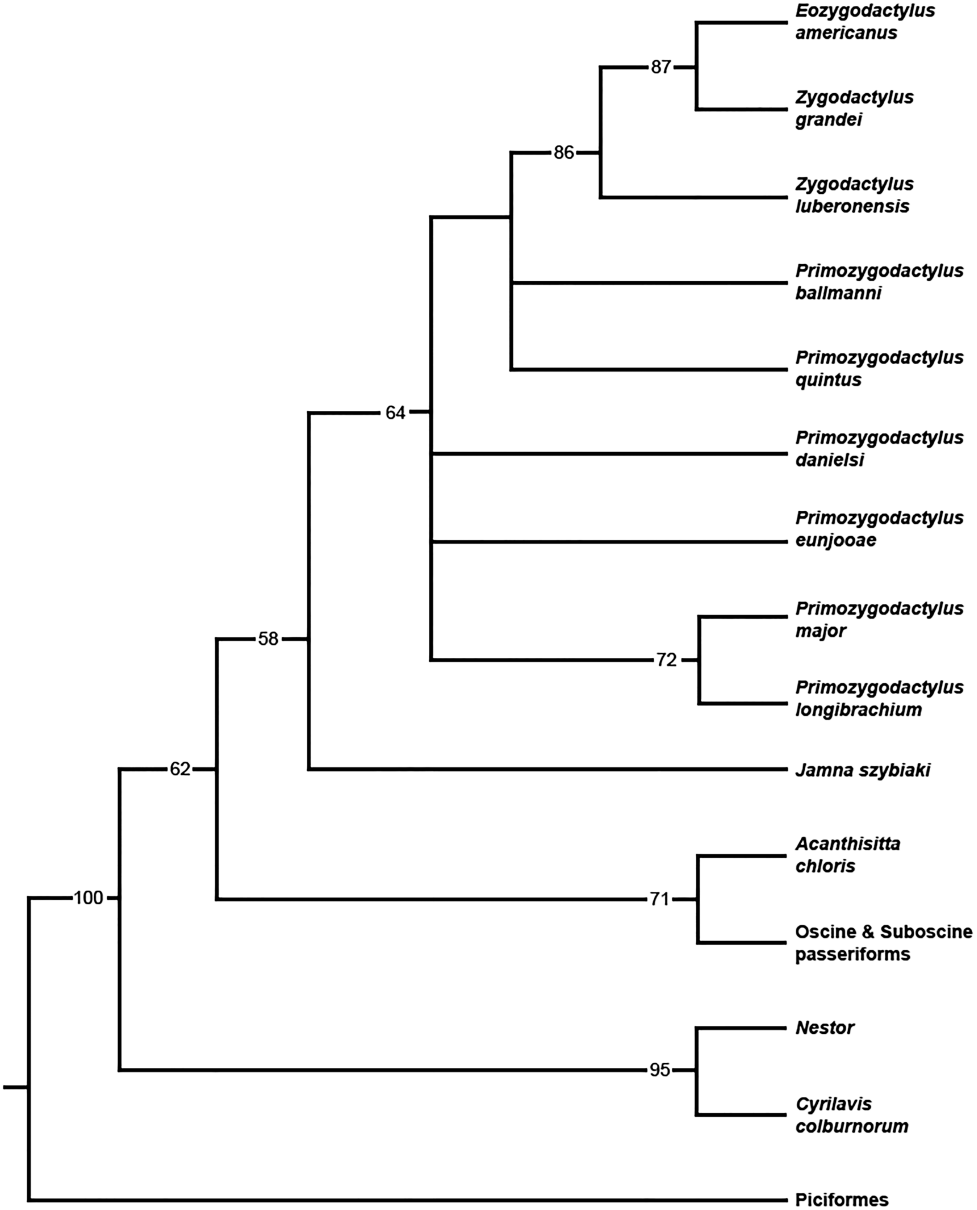
Phylogenetic hypothesis of Zygodactylidae. Strict consensus cladogram resulting from the branch-and-bound analysis of zygodactylid taxa (bootstrap values provided below associated nodes; MPTs = 15, TL = 103, CI = 0.76, RI = 0.65, RCI = 0.43).

When all 20 zygodactylid specimens scored for the phylogenetic analysis (Appendix I) were included, a completely unresolved polytomy was recovered (result not shown). Inclusion of significantly incomplete specimens resulting in terminals with few scored characters (e.g., *Z. grivensis*; missing data for 47 of 50 characters) served only to decrease resolution (i.e., operational taxonomic equivalents; Wiens, 2003). The subsequent analysis was restricted to the following nine ingroup taxa, represented by the name bearing holotype and paratype specimens: *E. americanus, Z. grandei, Z. luberonensis, P. danielsi, P. ballmanni, P. major, P. quintus, P. longibrachium*, and *P. eunjooae.* An analysis of the aforementioned zygodactylid taxa and all six outgroup taxa resulted in placement of a monophyletic Zygodactylidae as the sister taxon to the putative stem passeriform *J. szybiaki*, with Passeriformes, Psittaciformes, and Piciformes as successively more basal outgroups (Fig. 18). Paraphyly of Zygodactylidae with respect to sampled Pan–Passeriformes (i.e., *Jamna* + *Acanthsitta + oscine*, and *suboscine* passeriforms) requires only one additional step, supporting the previously hypothesized (Mayr, 2004, 2008, 2015) relatedness of passeriforms and zygodactylids. Groupings that place Piciformes or Psittaciformes as more closely related to Zygodactylidae than Pan–Passeriformes required at least three additional steps. *Z. luberonensis* is placed as the sister taxon to the clade composed of the two North American species *E. americanus* and *Z. grandei.* Species of *Primozydodactylus* are recovered in a paraphyletic assemblage to the exclusion of the *Zygodactylus* + *Eozygodactylus* clade. *P. quintus* and *P. ballmanni* are recovered in unreloved positions basal to the *Zygodactylus* + *Eozygodactylus* clade, with *P. eunjooae* and *P. danielsi*, as well as clade consisting of proposed sister *taxa P.* major *and P. longibrachium* in unresolved positions at the base of Zygodactylidae (Fig. 18). Consistent with analyses of molecular sequences (Hackett et al., 2008; Jarvis et al., 2014), *Acanthisitta* was recovered as the sister taxon to other passeriforms. The stem parrot *C. colburnorum* was recovered as the sister taxon to *Nestor*, the other sampled psittaciform (i.e., psittaciform and passeriform monophyly are supported).

## Discussion

### Anatomical insights

The previous referral of UWGM 40705 to *E. americanus* (Weidig, 2010) is not supported herein. Although UWGM 40705 is poorly preserved, a distinct dentiform process is visible on the carpometacarpus, which is present in *Z. grandei* and *P. minutus* (Mayr, 2008) but absent in the holotype specimens of *E. americanus* (Weidig, 2010) and *P. danielsi* (Mayr, 1998). UWGM 40705 has no scoreable characters in common with *Z. ignotus*. While an assignment to this taxon cannot be completely ruled out, *Z. ignotus* is from the Lower Miocene of Germany (∼18 Ma; Ballmann, 1969a). Thus, there is an approximately 30 Ma gap between the known stratigraphic ranges of these taxa, making assignment of UWGM 40705 to *Z. ignotus* highly unlikely.

FMNH PA 770, here referred to *E. americanus*, possesses the elongate, narrow pedal digits and weakly curved pedal unguals seen in *Z. grandei*, in the *E. americanus* paratype (Weidig, 2010), and *Z. luberonensis*. These specimens all exhibit the elongate tarsometatarsus and pronounced cnemial crests characteristic of all known zygodactylids. FMNH PA 770 exhibits a tibiotarsal length that is shorter than that of the holotype of *Z. grandei*, but consistent with the paratype of *E. americanus* (Table 4). Pedal digit lengths (Table 5) in the paratype of *E. americanus* and FMNH PA 770 are almost identical. FMNH PA 770 differs from *Z. grandei* and agrees with the *E. americanus* paratype in the ratio of the ungual of digit III to the sum of the length of the remaining phalanges of digit III (Appendix I, character 50). Referral of FMNH PA 770 to *E. americanus* is supported only if the referral of the paratype specimen to that species is valid. Therefore, in the absence of more complete specimens, the referral of FMNH PA 770 to *E. americanus* remains tentative.

UWGM 40363 and FMNH PA 757 preserve only cranial material, and due to similarities in beak size, narial construction and head shape with the holotype of *E. americanus* and that of *Z. grandei*, as well as similarities with *P. danielsi*, *P. ballmanni*, and *P. major*, they are here referred to as Zygodactylidae genus and species indeterminate. Narial opening size, beak length, and cranium length are nearly identical to the dimensions of the holotype of *Z. grandei* and holotype of *E. americanus* (Table 4). The similarity in the shape and size of these skulls is striking (Fig. 3). UWGM 40363 and FMNH PA 757 share with both the holotype of *Z. grandei* and the holotype specimen of *E. americanus* an approximately rectangular narial opening (Appendix I, character 1), a ventrally bowed jugal (Appendix I, character 3), and a narial opening greater than 50% of the length of the rostrum (Appendix I, character 2); all of these characters have a restricted distribution in Aves.

*Zygodactylus grandei* is the earliest known occurrence of *Zygodactylus*, which is previously known only from Oligocene and Miocene deposits in Europe. However, as *Zygodactylus* is recovered as paraphyletic with respect to *Eozygodactylus* in the phylogenetic results, it may be better said that sister taxa *E. americanus* and *Z. grandei* together comprise the earliest records this clade. *Eozygodactylus* may be best considered a junior synonym of *Zygodactylus.* However, although USNM 299821 and WDC-CGR-014 share a number of similarities with *Z. grandei*, phylogenetic analyses and other character data are consistent with *E. americanus* as a distinct, species-level taxon. For example, a dentiform process appears absent in the holotype and paratype of *E. americanus* and femoral measurements are distinct (in the paratype specimen) from *Z. grandei* (Table 4). The question remains whether these differences constitute generic level distinctions. Regardless, the nearly-identical skull morphology of the *E. americanus* holotype and *Z. grandei*, as well as the elongate tarsometatarsus, thin toes and weakly curved unguals of WDC-CGR-014, and the presence of an intermetacarpal process in all three specimens support the sister-taxon relationship between *Z. grandei* and *E. americanus* recovered herein. New material of *E. americanus* will be required to determine whether *Eozygodactylus* is a junior synonym of *Zygodactylus*. Regardless of generic changes, our results support the recognition of specimens currently referred to *Eozygodactylus americanus* as a distinct species.

As noted by Mayr & Zelenkov (2009), *Z. luberonensis* possessed proportionally longer legs than other Eocene zygodactylids, straighter ungual phalanges and a pedal digit III measuring only slightly less that the tarsometatarsus length. *Z. grandei* and the paratype of *E. americanus* have relatively straight pedal ungual phalanges and a pedal digit III/tarsometatarsus length ratio of 0.89 and 0.96, respectively. A ratio of 0.90 is seen in *Z. luberonensis* (Mayr, 2008). Further, as in *Z. luberonensis*, the trochlea accessoria is more bulbous than the trochlea accessoria of Pici or other zygodactylids. Thus, the expanded, posteriorly-extending trochlea does not necessarily represent, as Zelenkov (2007: 295) proposed, a “continuous evolutionary sequence” through time between *Primozygodactylu*s and *Zygodactylus*, but based on these new data, instead suggests that both tarsometatarsal modifications existed concurrently within Zygodactylidae. That is, rather than a morphocline observed to evolve over time, it appears that a broader range of foot morphologies were present in early Eocene zygodactylids.

As *Z. grandei* and *E. americanus* resemble *Z. luberonensis* in some limb proportions (Table 2), the bulbous accessory trochlea and weakly curved toes, it is possible that these taxa occupied a similar ecological niche. It has been suggested that the presence of elongate pedal phalanges and weakly curved unguals is correlated with primarily terrestrial taxa (e.g., sandpipers, roadrunners; Rüggerberg, 1960). Given the pronounced gracility and elongation of these taxa’s pedal phalanges, a more terrestrial ecology is a possibility. However, Mayr (2008) pointed out that a specialized food-manipulation use related to the highly modified accessory trochlea is a possible explanation for the zygodactyl condition. Regardless of its function in life, the specialized foot morphology of zygodactylids is distinctly different from that of passerines and other zygodactyl birds, including the Piciformes and Psittaciformes. The anatomical comparisons and phylogenetic results support this interpretation and suggest that the specialized foot morphology of these clades evolved independently (Mayr, 2009, 2015). There is no apparent morphocline displayed by zygodactylid taxa with respect to their foot specializations and additional evidence will be required to determine the full range of behaviors of which the feet of zygodactylids may have been capable. However, developmental evidence has suggested that anisodactyly may indeed be the derived state within Passeriformes, a hypothesis consistent with the zygodactyl condition of their proposed sister taxon, Zygodactylidae (Botelho et al., 2014). If correct, hypotheses of avian relationships (e.g., Hackett et al., 2008; Jarvis et al., 2014) that recover Psittaciformes as the sister taxon to Passeriformes would suggest that zygodactyly may indeed have a broader pleisiomorphic distribution than previously recognized (Botelho et al., 2014; Mayr, 2015). Furthermore, if avian toe orientation (i.e., anisodactyly versus zygodactyly in this case) is specified by developmentally controlled mechanical effects in ovo, such that only altricial birds can develop zygodactyl feet, then it follows that Zygodactylidae would have been altricial. The altricial development of passerines and parrots also provides some, albeit indirect, support for the hypothesis that zygodactylids were altricial.

### Zygodactylid diversity and biogeography

The description of *Z. grandei* confirms the previous estimate of North American zygodactylid species diversity (at least *n* = 2) proposed by Weidig (2010) and brings the total number of zygodactylid specimens known from North America to at least 8. However, additional species are likely represented by as yet undescribed material or even possibly the paratype of *E. americanus*. Although described alphataxonomic diversity of European zygodactylids (*n* = 8) outnumbers that of North America, the recognition of multiple North American species provides yet another example of similarity between the early Eocene avifaunas of the Green River Formation and Messel, Germany. Owing to the diverse avifauna described from the Green River Formation, taxa such as mousebirds (Coliiformes; Ksepka & Clarke, 2010a), rollers (Coracii; Clarke et al., 2009, 2014; Ksepka & Clarke, 2010b), and zygodactylids (Weidig, 2010), which were once considered to be exclusively Old World, are now known to have had much broader geographic distributions. Additionally, if Zygodactylidae are the sister taxon to Passeriformes as has been suggested (Mayr, 2008, 2009, 2015) and is supported by our phylogenetic results (Fig. 18), relatively coeval lineages of zygodactylids in North America and Europe provide additional context for interpretation of the growing number of Eocene and Oligocene records of Passeriformes (Boles, 1997a; Bocheński et al., 2011, 2013, 2014). If Zygodactylidae are in fact the sister taxon of Passeriformes (i.e., stem passeriforms), then the passerine total group had already achieved a relatively broad geographic distribution by ∼50 mya. Regardless of origination area, the presence of passerines and their stem group relatives in Europe, North America and Australia would suggest that dispersal between the northern and southern hemispheres had already taken place by the earliest Eocene, and that pre-Eocene fossil records of the groups’ dispersal should be sought after in Asia, Africa, Antarctica, and South America.

The recovery of *J. szybiaki* as the sister taxon to Zygodactylidae raises questions about the exact placement of this taxon within Pan–Passeriformes. Although this marks the first time that the systematic position of *J. szybiaki* has been evaluated in a phylogenetic analysis, the morphological characters scored for our analysis were designed to test the in-group relationships among species of Zygodactylidae (i.e., zygodactylid alphataxonomy), not broader relationships among taxa proposed as outgroups. Bootstrap support for the clade uniting *Jamna* and Zygodactylidae is also relatively low (Fig. 18). However, it is worth noting that minus the possession of a fossa in the distal metacarpal syntosis, the other characters cited as the basis for referral of *J. szybiaki* to Pan–Passeriformes by Bocheński et al. (2011) are variably present in known species of Zygodactylidae. Furthermore, the early Oligocene age of *J. szybiaki* is within the age range of Zygodactylidae (Eocene–Miocene) and it is not difficult to imagine a scenario in which multiple lineages of stem passeriforms co-existed in the Paleogene. However, we suggest that resolution of this issue will require denser taxon and character sampling than the analysis herein provides.

## Conclusion

The North American species *Z. grandei* and *E. americanus* were recovered as sister taxa to the exclusion of the European species *Z. luberonensis* in our phylogenetic results, suggesting that the synonymization of *Eozygodactylus* with *Zygodactylus* should be considered as additional specimens representing these and other potential *Zygodactylus* species are described. Our phylogenetic results bearing on the interrelationships among species of *Primozygodactylus* were not completely resolved. Thus, future recovery of additional specimens and or species of *Primozygodactylus* or other basal Zygodactylidae may facilitate resolution of these lingering systematic issues. It is notable that we find support for the previously proposed affinities of Zygodactylidae and Passeriformes (Fig. 18).

Developmental evidence supporting zygodactyly as the ancestral state in Passeriformes, the absence of a morphocline with respect to the specialized foot morphology of zygodactylids, and the proposed sister-taxon relationship between Passeriformes and Psittaciformes all suggest that zygodactyly may be the pleisiomorphic state for a broad clade including parrots, passerines, zygodactylids, and allies (Botelho et al., 2014; Mayr, 2015). Discoveries of Paleocene and Eocene passeriforms are needed to test this hypothesis, as the earliest passerine fossil foot is from the Oligocene and displays the anisodactyl condition characteristic of modern perching birds (Bocheński et al., 2014).

In the context of the proposed sister-taxon relationship between Zygodactylidae and Passeriformes, the presence of zygodactylid lineages in both Europe and North America has potential implications for the understanding of the physical and chronological biogeography of the passerine radiation—an active area of inquiry in studies of avian evolution. North American species of zygodactylids represent the oldest records of the clade and may provide a fossil calibration for avian divergence time analyses. Regardless of inferred systematic position, the description of *Z. grandei* sp. nov. greatly expands the geographic and age ranges of *Zygodactylus*. As with previously described fossil birds from the Green River Formation that have provided links between it and the Eocene laggerstäten of Messel, Germany (e.g., Clarke et al., 2009; Ksepka & Clarke, 2010a, 2010b), the shared zygodactylid diversity of Europe and North America provides yet another link between Cenozoic avifaunas from the Old and New Worlds.

## Appendix I: Morphological Character Descriptions and Scorings

### Morphological character descriptions

1. Skull: narial opening: shape: elongated (subrectangular) (0); short and subovoid (1). See Fig. 3.
2. Skull: narial opening: size: greater than 50% the length of rostrum (0); equal to or less than 50% the length of the rostrum (1). See Fig. 3.
3. Skull: jugal: shape: bowed ventrally (0); straight or concave (1). See Fig. 3.
4. Mandible: symphysis: length: (0) short (<1/5 length of mandible); (1) long (>1/5 length of mandible). Discussed by Weidig (2010; see Mayr, 2008, figure 2).
5. Mandible: mandibular fenestrae: absent (0); small (1); large (2). Character modified from that of Mayr (2015) and discussed by Mayr & Manegold (2006). See Fig. 3.
6. Presacral vertebrae: quantity: less than or equal to 19 (0); greater than 19 (1). Modified from character 8 of Mayr (2015).
7. Caudal vertebrae: pygostyle: shape: small with rounded dorsal margin (0); large, bladelike and dorsally projecting (1). Modified from character 7 of Mayr (2015). See Fig. 14.
8. Furcula: apophysis: absent or very small (0); well-developed and blade-like (1). Modified from character 10 of Mayr (2008). See Fig. 10.
9. Furcula, omal extremity: reduced, outline linear (0); wide, subtriangular omal extremity (1). Character 9 of Mayr (2004). Zygodactylidae is characterized by a wide subtriangular omal extremity (see figure 5c of Mayr, 2008).
10. Scapula: acromion: size: moderate (0); large (1). See figure 16 of Mayr (1998).
11. Scapula: acromium: shape: single process (0); bifurcated (1). Character 31 of Clarke et al. (2009). Also see figure 16 of Mayr (1998).
12. Coracoid: acrocoracoid process: not hooked (0); hooked (1). Discussed by Bocheński et al. (2011). See figure 2 of Mayr (2015).
13. Coracoid: procoracoid process: size: completely reduced (0); developed but moderate size, projecting approximately 1/4 the width of the coracoid shaft (1); large, projecting approximately half the width of the coracoid shaft (2); projecting more than 1/2 the width of the coracoid shaft (3). Ordered. Wording simplified and state (3) added from character 11 of Mayr (2004). State 0 is present in *Zygodactylus*. State 2 is present in *N. notabilis*. State 0 and 1 visible in Fig. 8.
14. Coracoid: medial side: flange absent, margin straight (0); flange present, margin convex (1). State 1 is present in *Primozygodactylus*, the holotype of *Z. grandei* (FMNH PA 726). Character noted by Mayr (2008). See Fig. 8.
15. Sternum: spina externa: bifurcation: not bifurcated (0); bifurcated (1). Discussed by Bocheński et al. (2011). See figure 24 of Mayr (1998).
16. Sternum: 2 notches (0); 4 notches (1). The presence or absence of medial sternal notches and trabeculae was discussed by Mayr (2015; character 15). See figure 24 of Mayr (1998).
17. Sternum: lateral trabeculae, posterior tip: extends posteriorly beyond tips of medial trabeculae (0); of same length or slightly shorter than medial trabeculae (1). State 0 is seen in the holotype of *E. americanus* (USNM 299821). State 1 is seen in the holotype of *Z. grandei* (FMNH PA 726).
18. Humerus: size of dorsal supracondylar process: absent or a very small protuberance (0); well-developed but not dorsally projected to a great degree (1); large, separated from shaft by a small notch (e.g., *Z. luberonensis*, *Z. grandei*, *E. americanus*; 2). Ordered. Noted by Manegold (2008).
19. Humerus: direction of dorsal supracondylar process: parallel to shaft of humerus (0); laterally directed in relation to humeral shaft (1).
20. Humerus: bicipital crest: size: moderate (0); large (1); exceptionally large, pointed (e.g., Psittaciformes; 2). Ordered.
21. Humerus:femur: relative length: femur longer than humerus (0); femur subequal to humerus (1); femur smaller than humerus (2). Ordered.
22. Humerus: *m. brachialis*: origin medially situated (0); laterally situated (1). State (0) is typical of Passeriformes, described zygodactylids, Piciformes and Coliiformes. Noted by Ashley (1941). Modified from character 16 of Mayr (2004).
23. Humerus: humeral head: narrow in posterior view (0); globose (1). Markedly more globose in *Z. luberonensis* than in *Z. grandei.*
24. Humerus: flexor process: short projection extending only slightly distal to the ventral condyle (0); markedly projecting distal to ventral condyle (1). State 0 is seen in Psittaciformes. State 1 is present in *Z. grandei*.
25. Ulna: feather papillae: absent (0); present (1).
26. Ulna: olecranon: short (0); long (1).
27. Ulna: relative length as compared with the tarsometatarsus: ulna shorter than tarsometatarsus (0); ulna subequal to tarsometatarsus (1); ulna longer than tarsometatarsus (2). Ordered.
28. Carpometacarpus: dorsal margin: protuberance mid-shaft (“dentiform process” of Mayr, 1998): absent, dorsal margin straight (0). present, protuberance mid-shaft (1). The holotype and paratype specimens of *E. americanus* have no mid-shaft protuberance, differentiating that specimen from *Z. grandei.* Character 46 of Clarke et al. (2009). See Fig. 7.
29. Carpometacarpus: intermetacarpal process: absent (0); present (1). Modified from character 47 of Clarke et al. (2009).
30. Carpometacarpus: intermetacarpal process: if present, unfused to minor metacarpal (as in Zygodactylidae; 0); fused to minor metacarpal (e.g., Passeriformes, Piciformes; 1). Modified from character 47 of Clarke et al. (2009).
31. Carpometacarpus: metacarpal III: somewhat curved, concave dorsally (0); straight (1).
32. Carpometacarpus: metacarpal III: subequal to metacarpal II (0); longer than metacarpal II (1). Modified from character 24 of Mayr (2004).
33. Manual digit III, phalanx 1: posterior margin: straight or slightly tapered (0); widened into a small tubercle (1). This character is present in both the holotype of *Z. grandei* (FMNH PA 726) and the holotype of *E. americanus* (USNM 299821), as well as in some Passeriformes (e.g., *Troglodytes aeclon*). Noted by Weidig (2010). See Fig. 7.
34. Manual digit I, phalanx 2: absent (0); present (1); State 1 found in Zygodactylidae.
35. Manual digit II, phalanx 1: smooth and linear ventrally (0); hooked ventrally, as in galbulids (1); convex ventrally, as in *Melopsittacus* (2). Modified from character 56 of Clarke et al. (2009).
36. Manual digit II, phalanx 2: >50% length of manual digit II, phalanx 1 (0); <50% length of manual digit II, phalanx 1.
37. Pelvis: obturator foramen: open (0); closed (1). This character is highly variable in Passeriformes and most Aves. Weidig (2010) stated that this is an apomorphy of *Eozygodactylus*.
38. Tibiotarsus: anterior projection of cranial cnemial crest: less than anteroposterior width of shaft of tibiotarsus (0); subequal to width of shaft of tibiotarsus (1); larger than width of shaft of tibiotarsus (2). Wording and number of states modified from character 39 of Mayr (2004).
39. Tarsometatarsus: anterior end of trochlea metatarsal IV: marked convexity on lateral tarsometatarsal margin: absent (0); present (1). This character was discussed by Mayr (2008).
40. Tarsometatarsus: a marked sulcus on the plantar surface of the proximal end of trochlea metatarsal IV bordering the lateral tarsometatarsal convexity: absent (0); present (1). (Fig 8; Mayr, 2008).
41. Tarsometatarsus: trochlea metatarsi II: plantarly directed projection: absent (e.g., Passeriformes); 0); present (e.g., Piciformes; 1). Character 40 of Mayr (2004).
42. Tarsometatarsus: shorter than humerus (0); subequal to humerus (1); exceeding humerus in length (2). This character is widely variable within Passeriformes, though the tarsometatarsus is uniformly longer than the humerus in Zygodactylidae. Ordered. Modified from character 34 of Mayr (2004).
43. Tarsometatarsus: posterior: metatarsal trochlea arranged such that plantar surface is concave (0); is aligned linearly, does not form concave plantar surface (1). State 0 is present in Zygodactylidae and Piciformes, and state1 is present in Coracii and Passeriformes. Commented on by Zelenkov (2007).
44. Tarsometatarsus: accessory trochlea: absent (0); present (1). Clarke et al. (2009), character 69. See figure 28 of Mayr (1998).
45. Tarsometatarsus: trochlea accessoria (if present): bulbous, reaches at least to middle of trochlea metatarsi III (e.g., *Zygodactylus*; 0); does not reach to trochlea metatarsi III (e.g., *Primozygodactylus*; 1). Noted by Mayr (2004).
46. Tarsometatarsus: metatarsal III: tubercle on lateral margin: absent (0); present (1). Character 59 of Mayr (2015).
47. Pedal digit II, phalanx I: less than 1/3 the length of pedal digit II, phalanx 2 (0); longer than 1/3 the length of pedal digit II, phalanx 2 (1). State 1 is an apomorphy for *Primozygodactylus danielsi*.
48. Pedal digit III: relative length as compared to the tarsometatarsus: pedal digit II ≥85% of tarsometatarsus length (0); pedal digit II <85% of tarsometatarsus length (1).
49. Pedal unguals: degree of curvature: slight to absent (0); moderate (1); pronounced curvature (2). Noted by Mayr (2008). State 0 is seen in *Zygodactylus*, and state 1 is present in *Primozygodactylus*. State 2 is seen in Passeriformes such as *A. chloris* and *T. migratorius*.
50. Ungual of pedal digit III: <25% of the sum of the length of the proximal three phalanges of digit III (0); ≥25% of the sum of the length of the proximal three phalanges of digit III (1). State 1 is autapomorphic for *Z. grandei* (FMNH PA 726) within Zygodactylidae.

**Table:** Morphological character matrix

Taxon	Specimen #	Character # 10	20	30	40	50
*Eozygodactylus americanus*	USNM 299821	0000?????00021?10210?10100?010011001??????????????				
*Eozygodactylus americanus*	WDC-CGR-014	???00????0?021?102100??10?001001?00?01???101?01000				
*Eozygodactylus americanus*	FMNH PA 770	??????0?????????????????0????????????1??1?010?1000				
*Zygodactylus grandei*	FMNH PA 726	0000?1???00021?112102?01000110111101?1111101001001				
*Zygodactylus grandei*	UWGM 40705	???????0?000?1?1121?????0??1101?1?????????????????				
*Zygodactylus luberonensis*	SMF Av 519	??000???10?0000??11?0111000110110100?111110?001000				
*Zygodactylus ignotus*	BSP 18164	???????????????????????????????1?????1?01?0100????				
*Zygodactylus grivensis*	FSL 151	??????????????????????????????????????????01?0????				
*Primozygodactylus ballmanni*	SMF ME 2108	000?0??????011???10111010?01??1?010?1000?101101110				
*Primozygodactylus danielsi*	SMF ME 2522	000?0???100011??1101110100111000010010001101100110				
*Primozygodactylus eunjooae*	SMF ME 1074	????????1????1???????1?10??1????0?0???000?01101110				
*Primozygodactylus major*	SMF ME 1758	000?0??111?011??110111010?2110??010?10000201101110				
*Primozygodactylus quintus*	SMF ME 11091	000??0??110011?1110111?1?00?1?100?00?????101??0?10				
*Primozygodactylus longibrach.*	SMF ME 11171	????????110011?????1??????2????????0?????2011?1111				
*Primoscens minutus*	BMNH A 4681	???????????????????????????110?0??????????????????				
Zygodactylidae indet.	UWGM 21421	?????????????????????????????????????2????01??1?00				
Zygodactylidae indet.	FMNH PA 757	000???????????????????????????????????????????????				
Zygodactylidae indet.	UWGM 40363	000???????????????????????????????????????????????				
Passeriformes SST		AA0AC10111A1001A0CA1D1A111C1110?00B112000A10?011C1				
*Acanthisitta chloris*		001?11011?001?11?11021?1110?111?A0??12000210?01120				
*Jamna szybiaki*	MSMD Av JAM-6	001?0?11???0?000?2002??101??110100?0?0????????????				
*Nestor* spp.		110??00001113000?0?2100011000?1?002?11001001011?2?				
*Cyrilavis colburnorum*	FMNH PA 754, 766	11100000000131?110?12000?0200?1000?0?0??100111?121				
Piciformes SST		1111111011101011111121011110110?00A?100010010011C1				

**Notes:**

Polymorphisms are coded as follows: “A” = 0/1; “B” = 0/2; “C” = 1/2; “D” = 2/3.

Missing data are indicated by “?.”

## Supplemental Information

10.7717/peerj.4950/supp-1 Supplemental Information 1 Morphological Character Scorings.

## Institutional abbreviations

BSP: Bayerische Staatssammlung für Paläontologie und Historische Geologie München, Germany
FMNH: Field Museum of Natural History, Chicago, Illinois, USA
FSL: Faculté des Sciences de Lyon, Lyon, France
HLMD: Hessisches Landes-Museum, Darmstadt, Germany
NAMAL: North American Museum of Ancient Life, Lehi, Utah, USA
SMF: Forschunginstitut Senckenberg, Frankfurt am Main, Germany
USNM: National Museum of Natural History, Smithsonian Institution, Washington, DC, USA
UWGM: University of Wyoming Geological Museum, Laramie, WY, USA
WDC: Wyoming Dinosaur Center, Thermopolis, WY, USA
WN: Private collection of M. Daniels, Clacton-on-Sea, England

## Additional Information and Declarations

### Data Availability

The following information was supplied regarding data availability: The nexus file with scorings for in-group and outgroup data is provided as a Supplemental File.

### New Species Registration

The following information was supplied regarding the registration of a newly described species: Publication LSID: urn:lsid:zoobank.org:pub:8F4D9D54-AFE5-4A79-8A49-FC804996DB5E; Genus name: *Zygodactylus*: urn:lsid:zoobank.org:act:5F621838-8105-44A4-ABEA-99FFC3DD835F; Species name: grandei: LSIDurn:lsid:zoobank.org:act:98880C5C-4150-4C6F-AE27-698D976EF2F0.
